# Promoting information technology for the sustainable development of the phosphate fertilizer industry: a case study of Guizhou Province, China

**DOI:** 10.1098/rsos.181160

**Published:** 2018-11-07

**Authors:** Shujie Ma, Zhibo Luo, Shanying Hu, Dingjiang Chen

**Affiliations:** 1 Department of Chemical Engineering, Center for Industrial Ecology, Tsinghua University, Beijing 100084, People's Republic of China; 2 Department of Resources and Environment Business, China International Engineering Consulting Corporation, Beijing 100048, People's Republic of China

**Keywords:** phosphate fertilizer, sustainable development, information technology, system dynamics, decoupling analysis

## Abstract

The information technology revolution has brought unprecedented opportunities to the sustainable development of the traditional phosphate fertilizer industry. In this paper, the changes in characteristic indexes during this technological progress and business innovation are investigated at the industrial level and for different stakeholders using scenario simulation analysis based on system dynamics. The results show that information technology will have a significant impact on the traditional fertilizer industry. The popularity of information technology represents a win–win situation for industries, farmers, enterprises and governments. The sustainable development of the phosphate fertilizer industry promoted by information technology means that agrochemical services are a new growth point for the industry, and farmers will be the largest beneficiaries. Enterprises will adjust their product structures to achieve the relevant phosphate reduction goals before 2020. At the government level, the indirect benefits from energy savings, water conservation and reductions in non-point source pollution control treatment also increase significantly. In the new production and sales model, the development of the phosphate fertilizer industry is completely decoupled from resource consumption. In the future, this technological progress will eventually form a sustainable network of industrial innovation patterns. Our finding suggests that the application of information technology in the phosphate fertilizer industry can stimulate the vitality of each entity in the industry and achieve a win–win situation.

## 1. Introduction

Phosphorus is an essential nutrient for living systems but is suffering from emerging sustainability challenges related to supply uncertainty and environmental pollution [1]. Large-scale mining of phosphate rock worldwide, largely for fertilizer production, has led to a massive acceleration in the phosphorus cycle [2]. China, with its rapid increase in population and affluence, is already the world's largest producer and consumer of phosphate fertilizer, but it faces perhaps the greatest sustainability challenges (such as a shortage of phosphorus resources, an irrational industrial structure, an imbalance between the supply and demand of the market and aquatic eutrophication) in its phosphorus industry [3–6]. These pressing sustainability issues highlight the importance of changing the current production and consumption patterns of phosphate fertilizer in China. Some studies depict sustainable phosphorus management as playing a key role in the production of fertilizer [7–9], agricultural activities (such as balancing fertilization, disposing of livestock excrement, adjusting livestock feed and changing the diet of residents [1,5,10–12]) and phosphorus recovery from waste [13–16]. However, these strategies are fine-tuned in the original production and consumption patterns of the phosphate fertilizer industry, and the application of new technologies, such as information technology, to explore innovations in the phosphate fertilizer industry is still at the exploratory stage. Formulating reasonable policies to encourage the phosphate fertilizer industry to combine with a new generation of information technology (such as the Internet and big data) is a new perspective that is of great concern to policy makers. For emerging paradigms such as information technology to be applied to traditional industries, especially in the absence of substantial reference cases, it is particularly important to quantitatively demonstrate their potential to achieve sustainable development and the implications for different stakeholders (including governments, industries, enterprise and farmers). To address this issue, we established a system dynamics (SD) model to assess the innovative effects of different applications of information technology in the phosphate fertilizer industry. In this paper, a general framework for applying information technology in traditional phosphate fertilizer industrial processes is first established. Thereafter, the SD model is applied to a typical scenario in Guizhou Province, China, with different development paths for the transformation modes (§2). By comparing different scenarios, the effects at the industry and enterprise levels can be identified, and the benefits to peasant households and the government are compared (§3). Finally, corresponding policy recommendations are proposed based on the results of different scenario analyses (§4). This work has important implications for promoting the transformation of phosphate fertilizer industry from a single chain system to a circular feedback system. This advancement in information technology will eventually lead to a network of industrial innovations. Additionally, this framework can be used to assess the effects of various policies and to support decision making for the promotion and application of new technologies, as well as the validation of environmental protection strategies.

## 2. Frameworks and methods

### 2.1. Innovative conceptual framework

The rapid development of information technology has provided significant strategic opportunities for the development of traditional industries, such as the phosphate fertilizer industry. The infiltration of information technology will lead to a subversive transformation of traditional fertilizer supply chain marketing. The construction of an Internet information sharing platform can effectively shorten the sales and service distance between fertilizer enterprises and farmers, and promote a new production–sales model, which then promotes innovation, the upgrading of agricultural services and diversification [17,18]. Innovations in fertilizer application will be combined with other technologies, such as irrigation and sowing in agricultural production, to improve the efficiency of planting production, conserve fertilizer resources, reduce water usage and reduce agricultural environmental pollution.

The Internet effectively promotes information matching, which enables a competitive business sales model that improves the efficiency of market information distribution and resource allocation. The concept of ‘Internet+,’ which plays a lead role in traditional industries, can also be applied to the production and supply of phosphate fertilizer. In the Internet+ thinking, the concept of being driven by fertilizer production enterprises, as the leading supply and marketing system, will be converted to a service-oriented application system, leading to the application of a farmer-oriented approach [19]. Fertilizer enterprises will be transformed from a simple production enterprise into service providers, whereas phosphate fertilizer products and agricultural services undergo common development. With Internet technology and Internet terminal products gradually spreading into rural areas, the Internet platform built by agricultural enterprises will gradually improve. An ‘Internet+ agriculture’ mode of formation will also lead to innovation in the fertilizer marketing model. This will effectively promote the opening of the production, supply and sales of fertilizer products. The agricultural service efficiency and quality, which will rely on the agricultural platform for online–offline integration, will also be improved.

**Figure 1.**
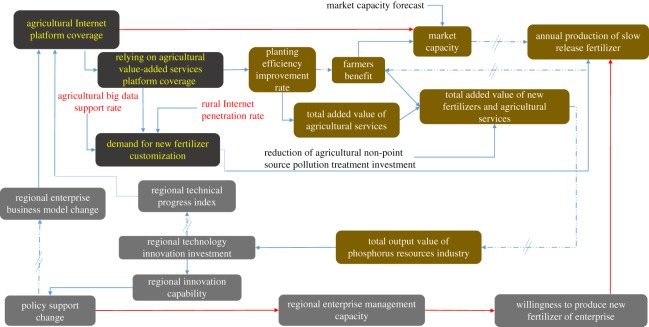
Innovative conceptual framework of the phosphate fertilizer industry. The solid module text and the plain text represent the state variables and auxiliary variables in the system dynamic (SD) model, respectively. A solid line represents a direct causal relationship between state variables, and a dotted line with a double slash indicates that the causal relationship between variables is indirect.

Relying on regional technological progress and a degree of change in the regional business model, combined with the application of Internet technology, we establish an Internet agricultural service platform. Changes in the coverage of this agricultural Internet platform can lead to improvements in agricultural value-added services and new fertilizer market demand, thereby improving the service efficiency and affecting the demand for slow-release fertilizer (figure 1). In the era of agricultural big data and the popularization of rural Internet, an agricultural value-added services platform based on the consumption of new fertilizer will increase the demand for customization. Differences in soil nutrient data, crop varieties and cropping patterns mean that the variety of fertilizers and the manner of their application can vary. Therefore, customized services are one direction of development for upstream fertilizer production. Fertilizer enterprises will respond to the specific requirements of farmers carrying out fertilizer production activities. In addition, the degree of customization of demand will be fed back to the production side and have a direct impact on the production of new fertilizers and their annual growth rate.

As shown in figure 1, farmers are at the heart of this transformation in the business model, enjoying gains from fertilizer savings, increased capacity, reduced labour costs and government subsidies. However, the price of new fertilizers is higher than that of conventional fertilizers, and farmers will need to bear some of the additional costs.

The development and scientific application of the new fertilizer market must be carried out by fertilizer enterprises. The main purpose of fertilizer enterprises, as an important driver of changes to the business model [20], is to maintain and expand the number of users (farmers) and open up new channels for fertilizer sales. The main benefits and motivation are the process by which new fertilizer becomes an alternative to the excess of traditional phosphate fertilizer, including the difference in the output value, an expansion of the market share and increases in the service output.

Under the guidance of the market economy, the government can benefit indirectly from changes to the business model. The main sources of benefit are reductions in the usage of natural resources and pollutant treatment costs, including the standard coal savings and water conservation, as well as agricultural non-point source pollution and a reduction in water pollution control costs.

### 2.2. System dynamics model structure

In the presence of multiple decision makers, and with the inherent complexity of supplier and consumer behaviour, feedback processes among modules, technological limitations and various kinds of delays, modelling the phosphate fertilizer industrial system is a complex problem. In the current methodology of studying the phosphate fertilizer industry, most research has focused on material flow analysis [21,22], stock analysis [23] and life cycle assessment [7]. However, these methods can only provide limited or partial research perspectives. For example, material flow analysis can only be used to present the flow and metabolism of phosphorus. Stock analysis is used to estimate the distribution of resources, and life cycle assessment is used to evaluate the environmental impact of the phosphate fertilizer from production to consumption. These methods cannot fully consider the complex relationship between phosphate fertilizer and other factors, especially the feedback between modules. Different from these methods, SD is a powerful methodology and modelling technique for understanding and exploring the feedback structure in complex systems [24]. Thus, SD is a suitable approach for modelling such complexities.

**Figure 2.**
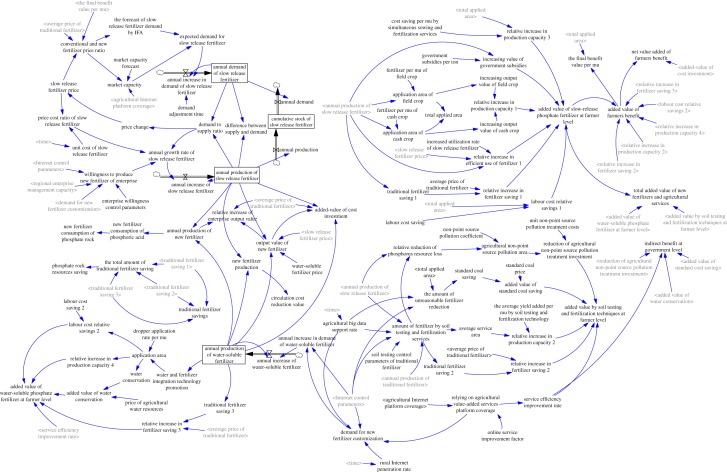
SD model of the phosphate fertilizer industry.

In a previous study, we developed an SD model of the regional phosphorus resource industry, covering resources, industry, economy, environment and society [25,26]. This model incorporated the market regulation and elimination mechanism, government support and the environmental constraints of the game structure and feedback structure between social innovation and industrial production. On this basis, we conducted a further study in which the Internet+ and big data technology were introduced into the phosphate fertilizer module (figure 2). We propose the transformation and upgrading of the consumption-side business model through innovation. A detailed causal analysis, simplified stock and flow diagrams (SFDs) (electronic supplementary material, figures S1–S13) and the functional relationships (electronic supplementary material, tables S1–S5) of each subsystem are available in the electronic supplementary materials file (S1). The data sources for the variables and parameters of the SD model are described in detail in tables S6–S12 (electronic supplementary material, S2). Moreover, the SD model validation and sensitivity analysis are presented in electronic supplementary material, S3. The system behaviour of the model output is consistent with the pre-existing causal mechanism (electronic supplementary material, figures S14–S16). Thus, the model can be applied in the subsequent strategy simulations.

### 2.3. Case study: phosphate fertilizer industry in Guizhou Province, China

Guizhou Province is located in southwest China (electronic supplementary material, figure S19). With the rapid development of thermal- and wet-process phosphoric acid technology in the phosphorus chemical industry, Guizhou Province has become a key phosphorus chemical resource base in China. At present, Guizhou Province is the most important production base in China for phosphorus fertilizer, yellow phosphorus and phosphoric acid. The histograms in electronic supplementary material, figure S19 represent phosphate rock production, phosphorus chemical industry sales, yellow phosphorus production and phosphate fertilizer production in China and Guizhou Province in 2014 (data are from the China Statistical Yearbook [27] and Guizhou Statistical Yearbook [28]). Guizhou Province accounted for 28.3%, 23.9%, 10.0% and 18.0% of these four indicators, respectively. Thus, we chose Guizhou Province as our research case and study area.

### 2.4. Scenario design

We considered traditional fertilizer production and traditional agricultural services as the baseline scenario. For comparison, we formulated the following scenarios: Internet + big data, Internet + big data + willingness of enterprise to produce new fertilizers, Internet + big data + willingness of enterprise to produce new fertilizers + traditional fertilizers for soil testing and fertilization (table 1). In analysing these scenarios, we investigated changes in the phosphorus resource industry and the effects on farmers, fertilizer enterprises, government behaviour and benefit distribution caused by changes to the business model. Electronic supplementary material, table S13 shows the range of parameters in different scenarios. The agricultural big data support rate and rural Internet penetration rate vary with time, ranging from 0 to 1. For the control parameters of the scenario simulation, a value of 0 indicates that the control is not present and a value of 1 indicates that the control is in effect.

**Table 1.** Strategy description and variable control for each scenario.

scenario	strategy	variable control
baseline scenario (S0)	traditional fertilizer production and traditional agricultural services	null
scenario 1 (S1)	Internet + big data	agricultural big data support rate, rural Internet penetration rate, agricultural Internet platform coverage, relying on agricultural value-added service platform coverage
scenario 2 (S2)	Internet + big data + willingness of enterprise to produce new fertilizers	agricultural big data support rate, rural Internet penetration rate, agricultural Internet platform coverage, relying on agricultural value-added service platform coverage, control parameter of willingness to produce new fertilizer of enterprise
scenario 3 (S3)	Internet + big data + willingness of enterprise to produce new fertilizers + traditional fertilizers for soil testing and fertilization	agricultural big data support rate, rural Internet penetration rate, agricultural Internet platform coverage, relying on agricultural value-added service platform coverage, control parameter of willingness to produce new fertilizers of enterprise, control parameters of traditional fertilizers for soil testing and fertilization

According to the actual situation, the rural Internet penetration rate and agricultural big data support rate from 2014 to 2025 are shown in electronic supplementary material, figures S17 and S18, respectively. ‘Demand for new fertilizer customization’ is an important part of the feedback link, mainly contributed by ‘Relying on agricultural value-added services platform coverage’, ‘Agricultural big data support rate’ and ‘Rural Internet penetration rate’. Regression analysis was conducted using SPSS software. The regression equation is presented in electronic supplementary material, table S13, and the variance analysis is given in electronic supplementary material, table S14.

### 2.5. Mathematical equation

#### 2.5.1. Output value of new fertilizer at industry level

The new phosphate fertilizers considered in this study are mainly water-soluble phosphate fertilizer and slow-release phosphate fertilizer. Therefore, the output value of the industry's new phosphate fertilizer is calculated as follows:

2.1 $$OV_{\mathrm{NPF}}=P_{\mathrm{WSPF}}\times Q_{\mathrm{WSPF}}+P_{\mathrm{SRPF}}\times Q_{\mathrm{SRPF}}\;,$$

where $OV_{\mathrm{NPF}}$ is the total output value of new phosphate fertilizer; $P_{\mathrm{WSPF}}$ and $P_{\mathrm{SRPF}}$ represent the price of water-soluble and slow-release phosphate fertilizers, respectively; and $Q_{\mathrm{WSPF}}$ and $Q_{\mathrm{SRPF}}$ represent the annual production of water-soluble and slow-release phosphate fertilizers, respectively.

#### 2.5.2. Relative increase in enterprise output value

By promoting new phosphate fertilizers, enterprises can obtain corresponding benefits from the production and sale process of the new product. To measure the growth in the output value of enterprises in the production and sale of new phosphate fertilizers, this study considers the relative increase in the enterprise output value. Based on the output value of enterprises in 2013, this is expressed as follows:

2.2 $$\mathrm{RIO}V_{\mathrm{En}}=OV_{\mathrm{En},i}-OV_{\mathrm{En},2013}\;,$$

where $\mathrm{RIO}V_{\mathrm{En}}$ is the relative increase in enterprise output value,$OV_{\mathrm{En},i}$ represents the output value of year *i* (where *i* is between 2014 and 2025), and$OV_{\mathrm{En},2013}$ represents the output value in 2013.

#### 2.5.3. Net added value of farmer benefits

The use of new fertilizers and fertilizer technology can bring additional benefits to farmers. The increase in farmer benefits can be expressed as:

2.3 $$\mathrm{AVFB}=\mathrm{AVF}B_{\mathrm{SRPF}}+\mathrm{AVF}B_{\mathrm{WSPF}}+\mathrm{AVF}B_{\mathrm{STFT}},$$

where AVFB is the added value of farmer benefits and$\mathrm{AVF}B_{\mathrm{SRPF}}$,$\mathrm{AVF}B_{\mathrm{WSPF}}$ and $\mathrm{AVF}B_{\mathrm{STFT}}$ are the added value of income at the farmer level on behalf of the slow-release phosphate fertilizer, water-soluble phosphate fertilizer and soil testing and fertilization techniques, respectively.

The added value of slow-release phosphate fertilizer at the farmer level and added value of water-soluble phosphate fertilizer at the farmer level are given by equations (2.4) and (2.5), respectively.

2.4 $$\mathrm{AVF}B_{\mathrm{SRPF}}=\mathrm{RIP}C_{1}+\mathrm{RIP}C_{3}+\mathrm{LC}S_{1}+\mathrm{IVGS}+\mathrm{RIEU}F_{1},$$

where $\mathrm{RIP}C_{1}$ and $\mathrm{RIP}C_{3}$ represent the relative increase in production capacity 1 and 3, $\mathrm{LC}S_{1}$ represents labour cost saving 1, IVGS represents the increasing value of government subsidies, and $\mathrm{RIEU}F_{1}$ represents the relative increase in the efficient use of fertilizer 1. The meaning of each term on the right of this formula is shown in the SFD diagram (electronic supplementary material, figure S4).

2.5 $$\mathrm{AVF}B_{\mathrm{WSPF}}=\mathrm{RIP}C_{4}+\mathrm{LC}S_{2}+\mathrm{RIF}S_{3},$$

where $\mathrm{RIP}C_{4}$ represents the relative increase in production capacity 4, $\mathrm{LC}S_{2}$ represents labour cost saving 2, and $\mathrm{RIF}S_{3}$ represents the relative increase in fertilizer saving 3.

In the application of new fertilizers, farmers need to buy new products, which will bring new costs. Therefore, the added value of the cost investment in this study is mainly derived from the cost of purchasing new fertilizers compared to that of conventional fertilizers.

The net AVFB can be calculated based on the direct AVFB and the added value of cost investment. This is calculated as:

2.6 NAVFB=AVFB−AVCI,

where NAVFB is the net AVFB andAVCI is the added value of cost investment.

#### 2.5.4. Indirect benefits at the government level

The application of soil testing and fertilization techniques and water and fertilizer integration technology not only improves the efficiency of planting, thereby increasing farmers' income, but also conserves water and coal resources and reduces water and soil pollution. Resource conservation and reduced environmental pollution control investment can be considered as an indirect increase in government revenue. Therefore, the indirect benefit at the government level is calculated as follows:

2.7 IBGL=AVSCS+AVWS+RAPTI×(1+SEIR),

where IBGL is the indirect benefit at government level;AVSCS and AVWS are the added value of standard coal savings and water conservation, respectively;RAPTI is the reduction of agricultural non-point source pollution treatment investment and SEIR is the service efficiency improvement rate.

Because the loss of phosphorus resources is reduced by scientific fertilization, the relative reduction of non-point source treatment investment can be converted to a reduction in the loss of phosphorus resources. The mathematical expression is:

2.8 IRAPTI=RAPA×UCPT

and

2.9 RAPA=RRPSL×NSPC ,

where RAPA is the reduction of the agricultural non-point source pollution area and UCPT is the unit cost of non-point source pollution treatment.RRPSL and NSPC are the relative reduction of phosphorus resource loss and the non-point source pollution coefficient, respectively.

Water and fertilizer integration technology will bring about an increase in the implementation of water conservation; the mathematical expression is as follows:

2.10 AVWC=80×TAA,

where TAA is the total applied area. According to the literature [29], water and fertilizer integration technology can bring about water savings of 80 m^3^ per mu (1 mu ≈ 667 m^2^).

The replacement of traditional fertilizers with new fertilizers will reduce the amount of synthetic ammonia, and scientific fertilization will reduce the amount of energy used in the fertilization process, which is equivalent to reducing the amount of standard coal. The equation for standard coal usage is:

2.11 AVSCS=AUFR×ASCCPT,

where AUFR represents the amount of unreasonable fertilizer reduction andASCCPT represents the amount of standard coal consumed per ton of unreasonable fertilizer application.

#### 2.5.5. Resource decoupling analysis

Decoupling is used to describe the relationship between economic growth and resource and environmental pressures. In a study on the relationship between carbon emissions and economic growth in Europe, Tapio [30] proposed the following equation to characterize the decoupling elasticity between CO_2_ emissions and GDP:

2.12 $$\varepsilon (CO_{2},\;\mathrm{GDP})=\;\frac{\Delta CO_{2}/CO_{2}}{\Delta \mathrm{GDP}/\mathrm{GDP}}.$$

In Tapio's study, decoupling is divided into three types: negative decoupling, decoupling and coupling. Furthermore, decoupling is broken down into weak decoupling (environmental pressure > 0, economic growth > 0, elastic coefficient 0 < *ɛ* < 0.8), strong decoupling (environmental pressure < 0, economic growth > 0, elastic coefficient *ɛ* < 0) and recessive decoupling (environmental pressure < 0, economic growth < 0, elastic coefficient *ɛ* > 1.2). In this study, we use the following form of the decoupling coefficient:

2.13 $$\begin{array}{rl} \varepsilon (\mathrm{PR},\;\mathrm{GIO})= & \frac{\Delta \mathrm{PR}/\mathrm{PR}}{\Delta \mathrm{GIO}/\mathrm{GIO}}\\ = & \frac{\Delta \mathrm{PR}}{\mathrm{PR}}*\frac{\mathrm{PCP}}{\mathrm{PCP}}*\frac{\mathrm{GIO}}{\Delta \mathrm{GIO}}\\ = & \frac{\Delta \mathrm{PR}}{\Delta \mathrm{GIO}}*\frac{\mathrm{PCP}}{\mathrm{PR}}*\frac{\mathrm{GIO}}{\mathrm{PCP}}\\ = & \frac{\Delta \mathrm{PR}}{\Delta \mathrm{GIO}}*\frac{\mathrm{PCP}}{\mathrm{PR}}*\frac{(\mathrm{GIO}(p)+\mathrm{GIO}(\mathrm{non}-p)+\mathrm{GIO}(\mathrm{as})+\mathrm{GIO}(\mathrm{el}))}{\mathrm{PCP}}\\ = & \frac{\Delta \mathrm{PR}}{\Delta \mathrm{GIO}}*\frac{\mathrm{PCP}}{\mathrm{PR}}*\left(\frac{\mathrm{GIO}(p)}{\mathrm{PCP}}+\frac{\mathrm{GIO}(\mathrm{non}-p)}{\mathrm{PCP}}+\frac{\mathrm{GIO}(\mathrm{as})}{\mathrm{PCP}}+\frac{\mathrm{GIO}(\mathrm{el})}{\mathrm{PCP}}\right). \end{array}$$

Based on Tapio's decoupling model, this study uses the resource decoupling coefficient to describe the relationship between the economic growth of the phosphorus resource industry system and phosphorus resource consumption. In equation (2.13), PR is the resource consumption of phosphate rock and GIO is the total output value of the phosphorus resources industry [31]. Equation (2.13) also includes PCP, which represents the output of phosphorus-containing resources (including yellow phosphorus production, low-concentration phosphate fertilizer production, high-concentration phosphate fertilizer production and phosphate production). We further consider the contribution of resource productivity [32] to the resource decoupling coefficient of each economic growth component. For example, GIO(p)/PCP represents the contribution of phosphorus products, GIO(non−p)/PCP represents the contribution of non-phosphorus products, GIO(as)/PCP represents the contribution of agricultural services and GIO(el)/PCP represents the contribution of pollutants to economic loss.

## 3. Results and discussion

### 3.1. Analysis of economic benefit

**Figure 3.**
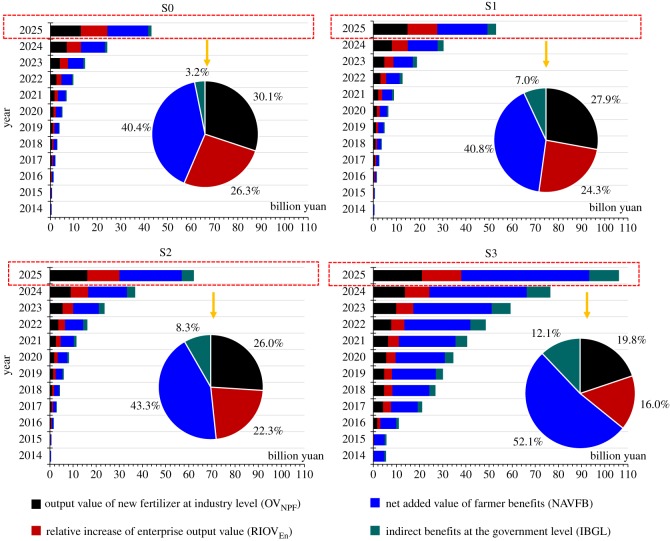
Comparison of the economic benefits of related subjects in different situations between 2014 and 2025. The histogram shows the economic benefits of four stakeholders. The pie chart shows the proportion of each output value of four stakeholders in 2025.

According to the mathematical equations derived in §2.5, we calculate the economic benefits to four different stakeholders: industry, enterprise, farmer and government. The results are shown in figure 3. In 2025, the combined economic benefits for all four entities under baseline scenario S0 and scenarios S1–S3 are 43.10, 53.03, 62.17 and 106.12 billion yuan, respectively. The total economic benefits in scenario S3 are approximately 2.5 times those in scenario S0. For the economic benefits of each subject, it is clear that the proportions change with the different scenarios, with farmer benefits accounting for the largest and most rapid growth. With the use of the Internet, the application of big data and the scientific application of traditional fertilizers (S3), the economic benefits received by farmers have expanded from 40.4% (S0) to 52.1% (S3). The increase in farmer benefits mainly comes from increases in the crop yield, reductions in fertilizer use, reductions in the labour force and increases in government subsidies. In terms of economic benefits, the next-best stakeholder is industry, followed by business and then government. As another beneficiary, the benefits to enterprises are derived from the sale of new fertilizers. As seen in figure 3, sales of new fertilizers will increase over those of traditional fertilizers with improvements in the Internet agricultural platform and the passage of time. Take scenario S3 as an example: following the conversion of fertilizer varieties, the enterprise output value increases from 0.09 billion yuan in 2014 to 16.97 billion yuan in 2025, a multiple of approximately 190. In general, the popularity of the Internet, the application of big data, the transformation of enterprise fertilizer production patterns and the scientific application of traditional fertilizers will create significant economic benefits for all four stakeholders. Further, as the core of the new business model, farmers will enjoy the greatest and most immediate benefits.

### 3.2. Changes in resource output at the industrial level

**Figure 4.**
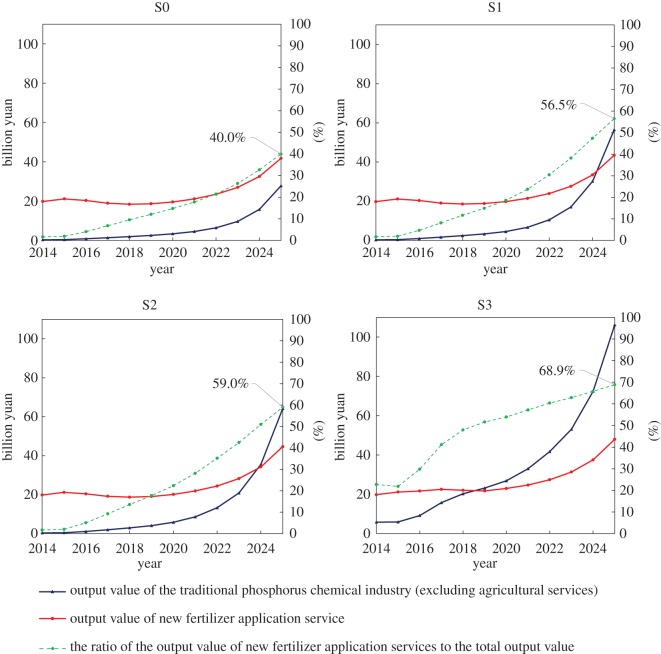
Comparison of the output value of the traditional phosphorus chemical industry and output value of new fertilizer application services under different scenarios.

Figure 4 shows the change in the output value of the traditional phosphorus chemical industry (excluding agricultural services), the output value of new fertilizer application services and the proportion of output value of new fertilizer application services to total output value between 2014 and 2025 under the four scenarios. The total output value is the sum of the output value of the traditional phosphorus chemical industry and the output value of the new fertilizer application services.

In scenario S0, the traditional phosphorus chemical industry (excluding agricultural services) increases from 19.82 billion yuan in 2014 to 41.79 billion yuan in 2025, and the output value of new fertilizer application services increases from 0.34 billion yuan in 2014 to 27.84 billion yuan. In scenarios S1, S2 and S3, the output value of the traditional phosphorus chemical industry increases slightly, but the total output value of new fertilizer application services exhibits significant growth. In the optimal scenario (S3), the total output value of new fertilizer application services reaches 106.19 billion yuan (2014 starting point of 5.83 billion yuan). This is because of the original traditional fertilizer application methods using soil testing and fertilization techniques since 2014. This scenario has the additional potential of substantial increases in soil testing and fertilization benefits. In this scenario, the proportion of new fertilizer application and services to total output (including the output value of the traditional phosphorus chemical industry and the output value of new fertilizer application services) is 68.9%, which proves that the new fertilizer application services have the widespread potential for promoting the development of the industry.

### 3.3. Benefit analysis of peasant households

**Figure 5.**
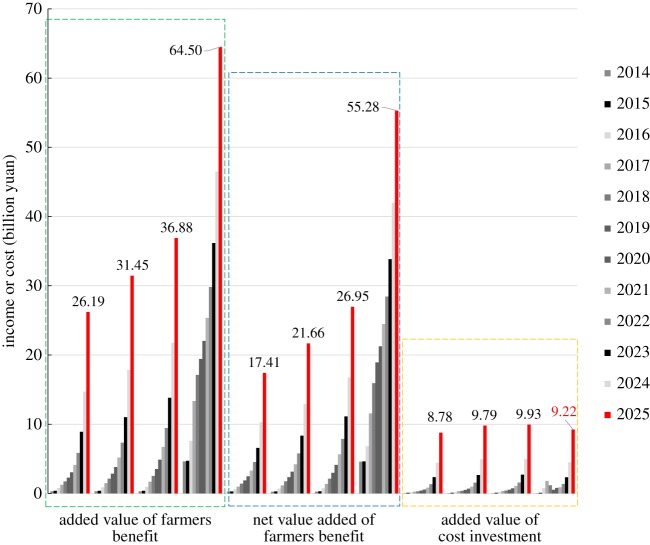
Comparison of the added value of farmer benefits and the added value of cost investment from 2014 to 2025 under four scenarios.

Figure 5 shows the AVFB and the added value of cost investment from 2014 to 2025 under the four scenarios. The bar graphs within the green, blue and orange dashed boxes represent the AVFB, the net AVFB (NAVFB) and the added value of cost investment of the farmer (AVCI), respectively. In each dashed box, the baseline scenario S0 and scenarios S1–3 run from left to right. As shown in figure 5, the AVFB and value of cost investment increase in all four scenarios, but the AVFB increases much more than the cost. Therefore, the net AVFB exhibits an increasing trend, which suggests that the new fertilization and business models will bring more benefits to farmers than the other inputs. In the case of replacement for fertilizer products purchased in 2025, the cost investment of farmers is approximately nine billion yuan, although the cost in S3 (9.22 billion yuan) is lower than in scenarios S1 (9.79 billion yuan) and S2 (9.93 billion yuan). This is because, under S3, the reduction in the price of new fertilizers and the widespread adoption of soil testing and fertilization services after 2020 will further reduce farmers' costs.

**Figure 6.**
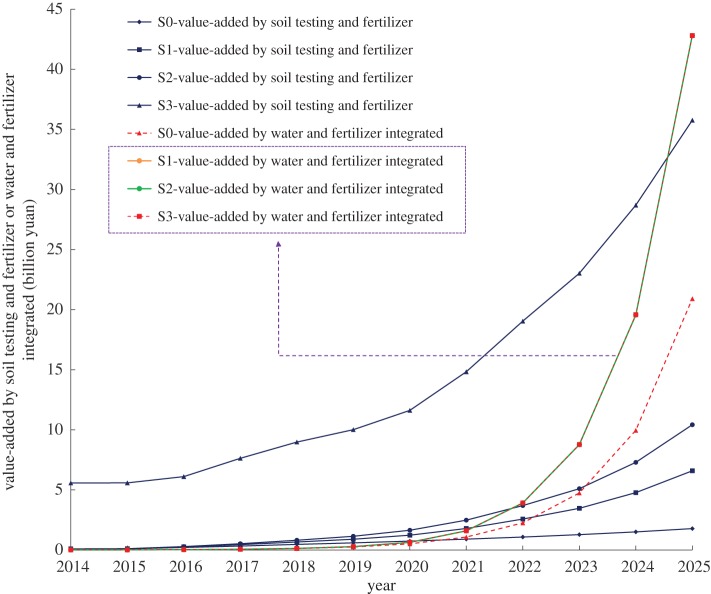
Added value of soil testing and fertilizer or water and fertilizer integrated in different scenarios. In scenarios S1–S3, the difference in the water and fertilizer integrated added value is very small, as shown by the purple dashed box.

New fertilizers and fertilization technologies have brought considerable benefits to farmers. The main fertilization techniques are soil testing, and water and fertilizer integration technology, which ensure the efficiency of fertilizer release. As shown in figure 6, the development of the Internet agricultural service platform enhances the added value of soil testing and fertilization techniques and water and fertilizer integration technology. In S3, traditional fertilizers were applied using soil testing and fertilization techniques, so the additional benefits were significantly higher than in the other scenarios. Compared to the soil testing and fertilization techniques, water and fertilizer integration technology is subject to the maturity of the dropper technology and the water-soluble fertilizer quality. Once the integration of water and fertilizer technology is mature, the increase in the added value of water and fertilizer integration technology is explosive (after 2022), reaching over 40 billion yuan in 2025. Therefore, it is clear that the added value from water and fertilizer integration technology was very small in S1–S3. This is because, in the Internet+ mode, water and fertilizer integration technology is more dependent on the new type of fertilizer production and demand for personalized field services. Therefore, the development trends are similar in these three scenarios. Overall, this is in line with the trend toward the development of customized fertilizers and field personalization services described in this study.

### 3.4. Changes in the mode of production at the enterprise level

**Figure 7.**
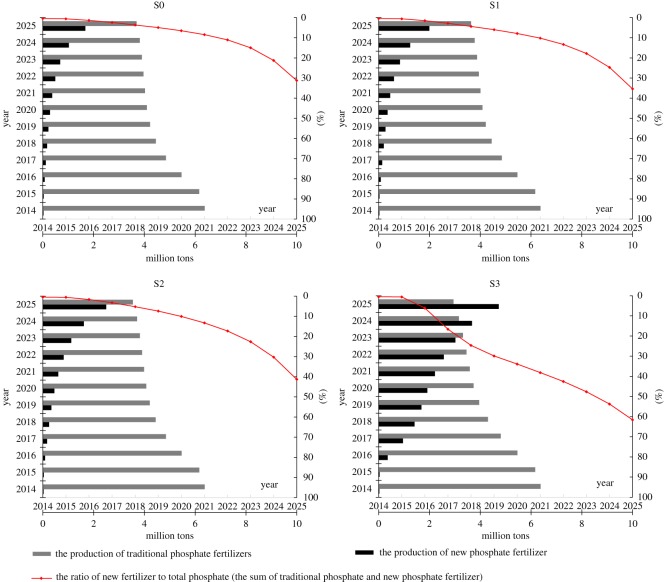
Comparison of the production of new phosphate fertilizer and traditional phosphate fertilizer.

With the improvement in farmer awareness of scientific fertilization, the demand for fertilizer has also changed. To comply with market demands, the enterprise production plan will require coordination between traditional phosphate fertilizers and new phosphate fertilizers. As seen from the single scenario in figure 7, the output of traditional phosphate fertilizers decreases year by year, whereas the output of new phosphate fertilizers increases year by year. With the new generation of information technology engendering deep changes in traditional modes of production, the proportion of new phosphate fertilizer products increases from 31.2% in S0 to 35.4% in S1, 41.3% in S2 and 61.6% in S3 by 2025. In S3, the production of new phosphate fertilizer (3.67 million tons) will exceed that of traditional phosphate fertilizer (3.16 million tons) by 2024. With this adjustment to the product structure, the proportion of high-end fertilizer varieties gradually increases, effectively changing the surplus situation of traditional low-end products. Overall, the total production of new phosphate fertilizers and traditional phosphate fertilizers first decreases, reaching a minimum in 2020 under all four scenarios, before increasing again. More importantly, the total output of phosphate fertilizer in the four scenarios in 2020 (4.39, 4.44, 4.53 and 5.66 million tons, respectively) is less than that in 2014 (6.41 million tons), indicating that the goal of reducing fertilizer production has been achieved. As a result of the higher resource productivity of new phosphate fertilizers, the consumption of phosphate rock resources will be lower than that of traditional low-end phosphate fertilizer products. Coupled with the reduction in the total output of phosphate fertilizers, the phosphate rock resources will lead to further savings. As a producer of fertilizer products, the enterprise not only adjusts its product structure but also adds value by changing the original production mode according to market changes.

### 3.5. Indirect benefit analysis at the government level

**Figure 8.**
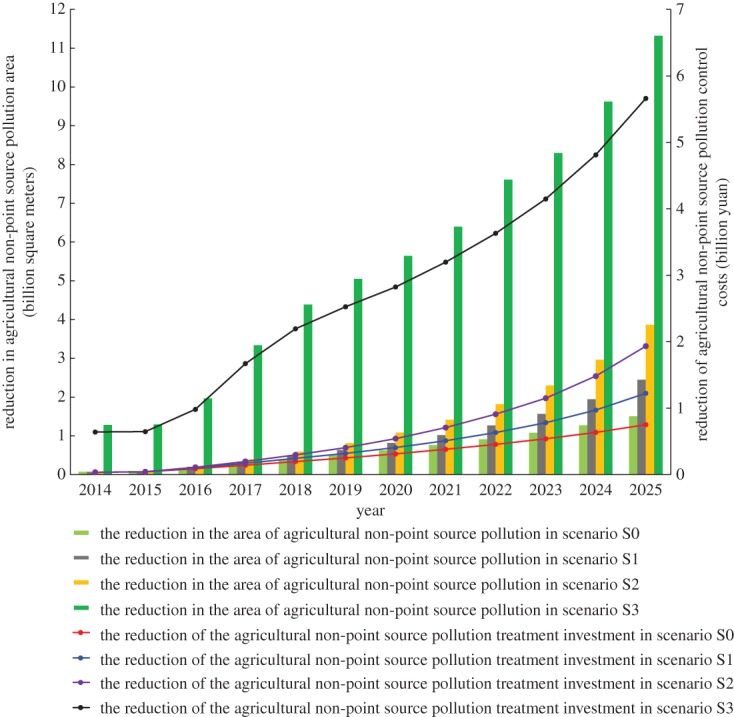
Reduction of agricultural non-point source pollution area and treatment investment under different scenarios.

An indirect benefit analysis at the government level is shown in figure 3. We can clearly see that the best-case scenario for indirect benefits at the government level occurs under S3, reaching approximately nine times that of scenario S0 in 2025. Among the indirect benefits at the government level, the largest proportion is the reduction in pollutant treatment investment. In addition, agricultural non-point source pollution in China has aroused widespread concern, so this study focuses on agricultural non-point source pollution problems. Figure 8 shows that, in scenarios S1–S3, the reduction in the area of agricultural non-point source pollution accelerates year by year, especially in scenario S3, where the decrease is most obvious. Thus, the area of agricultural non-point source pollution decreases year by year. In the optimal scenario (S3), the value of this reduction in non-point source pollution reaches 11.32 billion m^2^ in 2025, and the reduction in treatment investment reaches 5.66 billion yuan. Under this scenario, the agricultural non-point source pollution can be effectively controlled, significantly reducing the costs of government pollution control.

As well as the reduction in agricultural non-point source pollution treatment costs, standard coal savings and water conservation also contribute to the indirect benefits at the government level. Compared to the baseline scenario (S0), water conservation in scenarios S1–S3 increases from 0.80 million tons in 2014 to 3.02 billion tons in 2025 (electronic supplementary material, figure S20). This is because water and fertilizer integration technology can effectively reduce water consumption, and the technology is applied in all three scenarios at the same time. The replacement of traditional fertilizers with new fertilizers and new fertilization processes will reduce the standard coal usage. In the best scenario (S3), the standard coal savings are 0.60 million tons in 2025, corresponding to an increase in coal savings equivalent to 397.07 million yuan (electronic supplementary material, figure S21). With the increase in indirect benefits to the government, the deeper meaning is resource conservation and environmental protection. Therefore, the promotion and application of a new generation of information technology have significant social benefits.

### 3.6. Resource decoupling analysis

The resource decoupling coefficients, calculated from equation (2.13), for each scenario between 2014 and 2025 are presented in table 2. The resource decoupling coefficients are less than zero in all four scenarios, indicating that the development models for each scenario exhibit strong decoupling. However, from the baseline scenario S0 to scenario S3, the value of ɛ gradually increases, and the value for S3 is close to zero. In the consumption-side innovation pattern, the degree of demand for products and the dependence on resources increase with rises in product conversion and downstream demand. The degree of decoupling under this high-level scenario analysis exhibits a downward trend. Therefore, while actively exploring new products and models, the production of new products needs to be controlled, and phosphate rock consumption needs to be monitored in real time. In general, it is important to maintain a balance between production and consumption.

**Table 2.** Resource decoupling coefficients for the four scenarios between 2014 and 2025.

scenario	*ɛ*(PR, GIO)
S0	−0.0303
S1	−0.0169
S2	−0.0133
S3	−0.0086

Furthermore, the contribution of each component of economic output is analysed in table 3. The contribution of phosphorus products, non-phosphorus products, agricultural services and economic loss increases from 2014 to 2025 in the baseline scenario S0 and scenario S3. By 2025, the total amount under S3 has increased by more than double that in S0. The most obvious contribution is from agricultural services. In the baseline scenario, phosphorus products contribute 59.5% to economic output, somewhat greater than the 39.9% of agricultural services. In S3, the agricultural services contribution is 69.0%, which is far greater than the 30.7% contribution from phosphorus products. This contribution from agricultural services effectively alleviates the pressure of the economic growth of the phosphorus resource industry on resources and the environment.

**Table 3.** Contribution and proportion of economic output of unit resource under scenarios S0 and S3 (yuan/ton).

scenario	year	category	GIO(p)/PCP	GIO(non−p)/PCP	GIO(s)/PCP	GIO(el)/PCP	total value
S0	2014	contribution value of component	1702.80	24.20	35.04	−48.79	1713.25
		proportion of contribution	99.4%	1.4%	2.1%	−2.9%	100.0%
	2025	contribution value of component	3795.90	72.65	2547.10	−33.72	6381.93
		proportion of contribution	59.5%	1.1%	39.9%	−0.5%	100.0%
S3	2014	contribution value of component	1702.80	24.20	493.60	−48.79	2171.81
		proportion of contribution	78.4%	1.1%	22.7%	−2.3%	100.0%
	2025	contribution value of component	4205.30	80.37	9460.17	−27.06	13718.78
		proportion of contribution	30.7%	0.6%	69.0%	−0.2%	100.0%

## 4. Conclusion

Rapid advances in information technology have led to the opportunity for the sustainable development of traditional industries. This paper has focused on the excess capacity of the phosphate fertilizer industry and external technological impact of the phosphorus resource industry, with particular emphasis on the use of business model changes on the consumption side leading to industrial innovation.

This study considered the new generation of information technology, such as the Internet+ and big data, and investigated the production of new high-end fertilizers and the establishment of an Internet sales service platform through a series of SD simulations. The results show that, under the optimal scenario (S3), profits from new services exceed the output value of traditional products by up to 56.5%. Farmers gain the most benefit in this scenario. In the application of new fertilizer technology, farmer benefits increase with the degree of innovation, but the input cost depends on the popularization of the rural Internet and improvements in the cultural level of farmers. In the case of business model changes, enterprises are expected to adjust their product structures. The volume of fertilizer production is expected to be lowest in 2020, and to meet the goal of reductions in phosphate fertilizers. To meet the new demands of farmers, the production mode of new fertilizer products has shifted to customization of demand. In the open innovation mode, the proportion of new phosphate fertilizers reaches 61.6%. In the scenarios examined in this paper, the government also significantly benefits from savings in standard coal, water conservation and non-point source pollution control treatment investment. The industry achieves complete decoupling in the new business model, but the degree of decoupling declines as the high-level processes become more prevalent. Therefore, controlling the potential risks of new production and marketing patterns requires careful consideration.

In terms of the sustainable development of intelligent agriculture, farmer demands for fertilizer customization will gradually increase. Production and consumption patterns will determine the extent of this process. Industrial development will place more emphasis on the consumption side of individual needs, whereas the fertilizer production side will pay more attention to feedback from the consumer side through product formula adjustment, technical guidance, and other specific requirements. In the industry chain, including network nodes from fertilizer manufacturing to circulation, the industry will transform from a series of one-way chain activities into circular feedback activities. With new business models and new technologies continuing to emerge, the system will grow to include multi-directional industrial creation and innovation activities, ultimately forming a network of industrial innovation. This study not only has a significant impact on changing, refactoring and even subverting the traditional business model of the phosphate fertilizer industry but could also inspire a holistic approach to sustainable development in the phosphorus resource industry, the green development of agriculture, nutrient management and the application of new technologies. Overall, the modelling approach and framework presented in this paper can function as a decision support tool for other nutrient-intensive regions.

## Supplementary Material

Supplementary Materials

## Data accessibility

The datasets supporting this article have been uploaded as part of the electronic supplementary material.
