# Supplementary material for: Promoting information technology for the sustainable development of the phosphate fertilizer industry: a case study of Guizhou Province, China
Source: R Soc Open Sci. 2018 Nov 7;5(11):181160. doi: 10.1098/rsos.181160 (PMC6281935; doi:10.1098/rsos.181160)
Supplement: Supplementary Materials [file rsos181160supp1.pdf]

Royal Society Open Science

Supplementary Materials

Promoting information technology for the sustainable development of the phosphate fertilizer industry: A case study of Guizhou Province, China

Shujie Ma [1,2,#], Zhibo Luo [1,#], Shanying Hu [1,*], Dingjiang Chen [1]

1 Center for Industrial Ecology, Department of Chemical Engineering, Tsinghua University, Beijing 100084, China;
2 Department of Resources & Environment Business, China International Engineering Consulting Corporation, Beijing 100048, China
# These authors contributed equally to this work.
* Correspondence: hxr-dce@tsinghua.edu.cn; Tel.: +86-10-62794513

The main purpose of this appendix file is to describe the modeling process of the system dynamics (SD), explain and document the causal relationships of the variables and Stock and Flow Diagrams (SFDs) of each subsystem and the values and rationale (e.g., equation, parameter, and activity data) used to calculate the SD model [1, 2]. Historical data for the main parameters of each subsystem, relevant technical parameters and the necessary model validation and sensitivity analysis of SD model are also demonstrated in this section.

1 Model structures and settings

1.1 Resource subsystem

1.1.1 Causal analysis of resource subsystems

In this study, a resource subsystem involves exploration, mining, mineral processing and so on. During mine exploration and rock selection, exploration and mining growth rates are two important indexes that affect increases or decreases in ore stocks. However, with phosphate resource shortages and the unsustainable exploitation of phosphorite, exploration growth has declined while phosphorus ore exploitation with downstream ore processing and market demand have increased each year. In the process, the ore recovery rate and the recovery rate of mineral processing are two important indexes reflecting scientific and efficient processes of ore exploitation and selection. However, with shortages of PR resources and unsustainable development, the exploration growth rate of PR has declined each year, and annual mining levels have also increased annually with downstream ore processing and the

expansion of market consumption demand. In this process, the ore recovery rate and ore dressing recovery percentage are two important factors that reflect whether mining and dressing processes are scientific and efficient. Therefore, mainly mining and recovery processes are examined in this study. Part of the examined phosphate is used for local consumption while remainder is transported out of the region, which should have certain effects on the regional economy.

As a result of the over-exploitation and inappropriate utilization of high-grade PR, the extensive exploitation of low-grade PR, the formation of resource waste and environmental pollution resulting from downstream processing and PR use, the ecological environment of the mining and processing area has become increasingly vulnerable. Therefore, we establish resource and environmental carrying capacities to characterize direct responses of mining environments to resource development and utilization.

The resources and environment carrying capacity value denotes the cumulative change in the carrying capacity of resources and the environment. Changes in the carrying capacity of resources and the environment are affected by mining activities levels, ore recovery rates, an annual increases in the resource environmental impact coefficient [3]. An increase in annual exploration denotes the presence of more resources and thus more positive impacts on the resources and environment carrying capacity. In contrast, when the annual level of mining increases each year, this will increase the resources and environmental carrying capacity, and with an increase in the resource environmental impact coefficient, the resulting dual effect will further weaken the regional carrying capacity of resources and the environment.

1.1.2 The SFDs of the resource subsystem

According to the resource subsystem causality analysis shown in Section 1.1.1, SFDs of the resource development subsystem are shown in Figure S1.

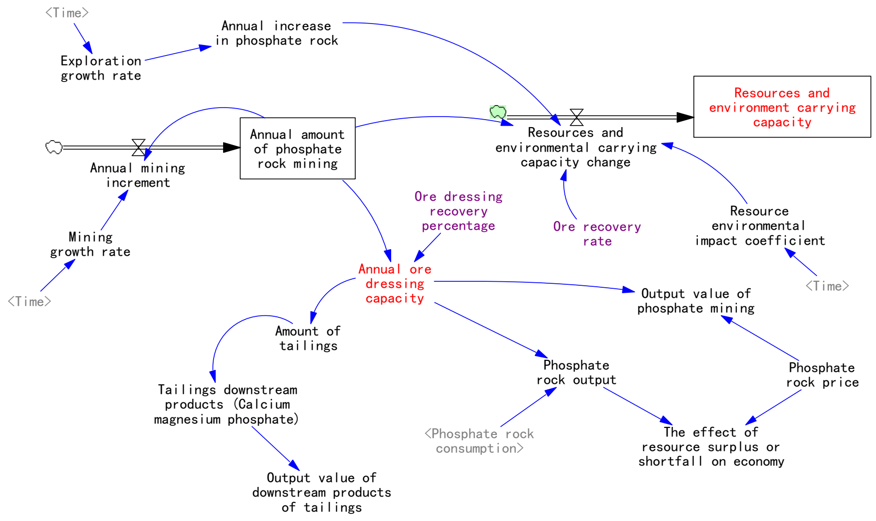

Fig. S1 SFDs of the resource subsystem.

At present, tailings produced by the ore dressing in the phosphate production area are treated by stockpiling and backfilling [4], resulting in the production of resource waste and environmental pollution. Therefore, we propose the reutilization of tailings as an upgrade measure, and especially for the processing of low-grade tailings. Tailings contain a small amount of phosphorus and several magnesium elements and can be used to produce calcium and magnesium fertilizers as well as other alkaline fertilizers, magnesium and calcium chemicals [5]. In this model, magnesium and phosphorus in the tailings are used to produce calcium magnesium phosphate.

1.1.3 The main variables and function relations in resource subsystem

The resource subsystem contains 20 variables, including 2 state variables, 2 rate variables, and 16 auxiliary variables. The equations between the main variables are shown in Table S1.

Table S1 Main variables and their equations in resource subsystem.

Variable	The type of the variable	Unit	Equation
Annual amount of mining	State variable	Ton	Annual amount of mining = INTEG (Annual mining increment, Initial value of mining)
Annual ore dressing capacity	Auxiliary variable	Ton	Annual ore dressing capacity = Annual amount of mining * Ore dressing recovery

			percentage
Resources and environment carrying capacity	State variable	Ton	Resources and environment carrying capacity = INTEG (Resources and environmental carrying capacity change,8.4332e+008),
Resources and environmental carrying capacity change	Rate variable	Ton /year	Resources and environmental carrying capacity change = Annual increase in * Ore recovery rate - Annual amount of mining *(1+ Resource environmental impact coefficient)
Exploration growth rate	Auxiliary variable	Dmnl	0.15*EXP(-0.15*((Time- Initial year)+1))
output	Auxiliary variable	Ton	output = Annual ore dressing capacity - consumption

1.2 Industrial Subsystem

1.2.1 Causal analysis of the industrial subsystem

In regards to industrial processes, the phosphorus chemicals industry applies two types of processes: wet process phosphoric acid production (WPPAP) and thermal process phosphoric acid production (TPPAP). However, through the upgrading of phosphate fertilizer products and the substitution of WPPAP for TPPAP, we establish a new industrial chain of fertilizer production [6] and of industrial grade phosphoric acid (IGPA) downstream products [7] on the basis of traditional WPPAP. Therefore, the industrial subsystem includes four modules: the traditional WPPAP module, the traditional TPPAP module, the new fertilizer module and the IGPA downstream module.

The traditional WPPAP module mainly involves the production and processing of low-concentration phosphate fertilizer (LCPF) and high-concentration phosphate fertilizer (HCPF). The traditional TPPAP module involves phosphorous acid (PA) deep processing and phosphoric acid fine processing. Products generated through TPPAP include yellow phosphorus (YP) and downstream phosphates. New fertilizer modules involve the production and application of new varieties of PF. IGPA downstream modules involve downstream high-end phosphate production based on WPPAP purification technologies [8].

Due to the serious unsustainability of China's PF industry, PF production control is key to industrial transformation and upgrading. The production and supply of PF products belongs to the supply chain of the manufacturing industry, and its characteristics are consistent with basic characteristics of the supply chain. Therefore, based on the basic model of inventory control and principles of material delay and information smoothing in the feedback loop, we present a supply and demand causality diagram of PF based on the actual production and sale of PF. By forecasting downstream market demand, the production demand for phosphate compound fertilizer can be obtained and the expected annual output of PF can be deduced. According to actual conditions, it is necessary to set a reasonable moving average time and an expected stock and stock adjustment time. As demand for PF production directly affects the price of PF product changes, set price fluctuations associated with the adjusted price will be re-applied to the proportion of PF demand. In addition, price changes directly affect the production efficiency of enterprises. In cases involving excess phosphate, the price of a direct impact on production capacity fluctuations in increase and elimination rates and the production capacity of the elimination rate directly affect the expected output of PF products.

1.2.2 The SFDs of an industrial subsystem

From the above causal analysis, SFDs of the four modules in an industrial subsystem are shown in Figure S2-5.

The traditional WPPAP module takes into account downstream phosphorus and WPPAP stock control. The main products of the TPPAP module are YP products and downstream phosphorus products of YP. The annual growth rate of YP is controlled by the investment growth rate coefficient, and PR consumption is reflected by the YP consumption coefficient.

With the upgrading of products, new fertilizers, such as slow controlled release fertilizer (SCRF) and water-soluble fertilizer, gradually replace the traditional fertilizer. Under the traditional mode, the application of new fertilizers was found to be rare. Under the transition mode, the production and application of new fertilizers was found to be relatively effective. However, due to the relatively high price of new fertilizers at initial stages, farmers have been slow to adopt them.

Through the progression of WPPAP purification mechanisms; downstream WPPAP products, such as IGPA; and food grade phosphoric acid production [7], demands will continue to increase. The development of electric vehicles will promote the development of phosphorus-based battery materials. Lithium iron phosphate for positive battery electrodes [9] and lithium hexafluorophosphate for battery electrolytes [10] have a faster growth rate. Due to bromine flame retardant pollution problems, phosphorus-based flame retardants [11], as a result of brominated halogen flame

retardant substitution, will become a trend, and we thus used ammonium polyphosphate (APP) as a representative phosphorus-based flame retardant. Mono-calcium and di-calcium phosphate (MDCP), a new variety of phosphorus feed [12], is an environmentally friendly product. While the mainstream use of dicalcium phosphate (DCP) in China will also have alternative effects, at the same time, monocalcium phosphate (MCP) and tricalcium phosphate (TCP) also occupy a small part of the market share. With the rapid development of electronic products, domestic electronic grade phosphoric acid will spur WPPAP purification technology breakthroughs and scale increases.

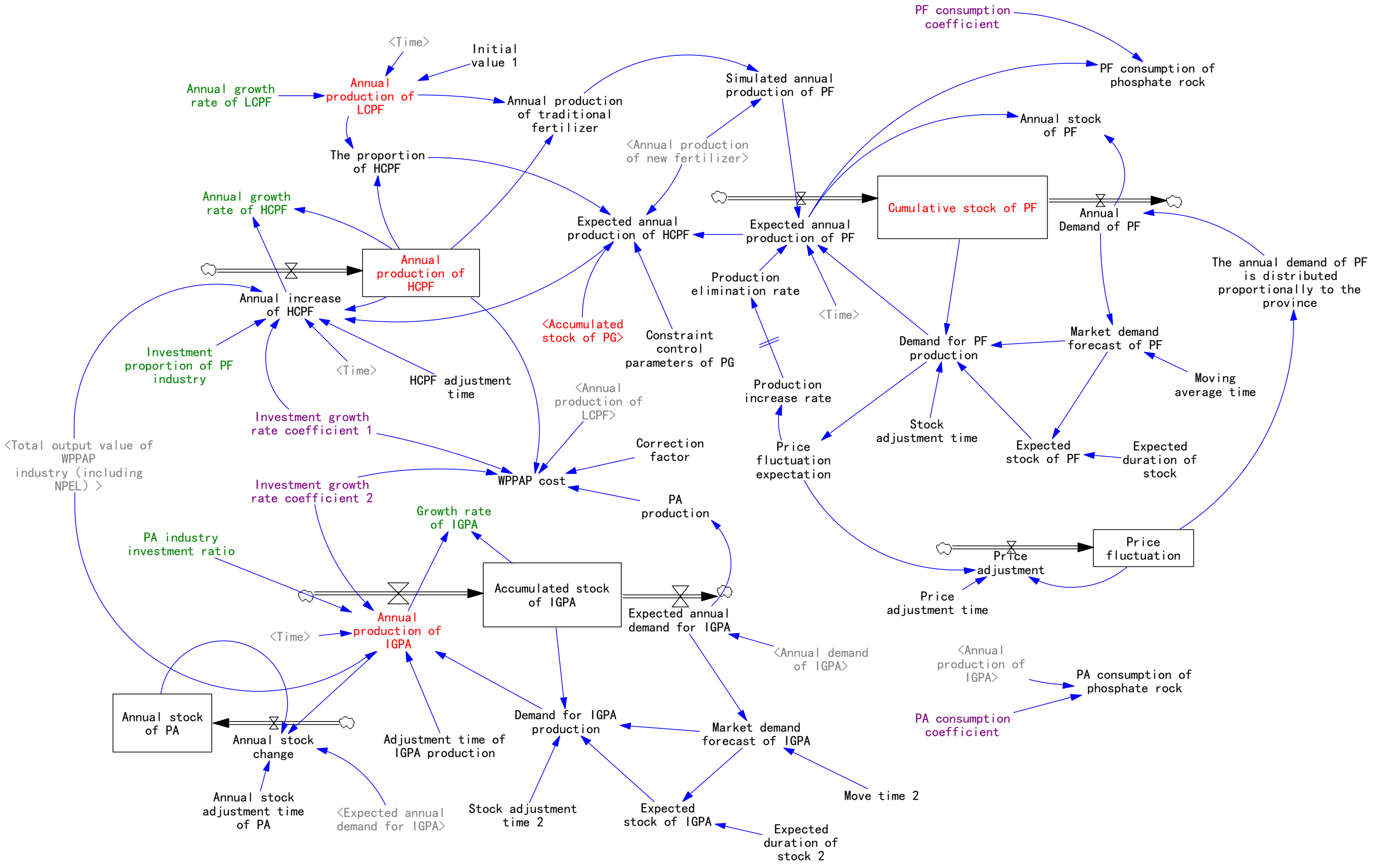

Fig. S2 SFDs of the traditional WPPAP module.

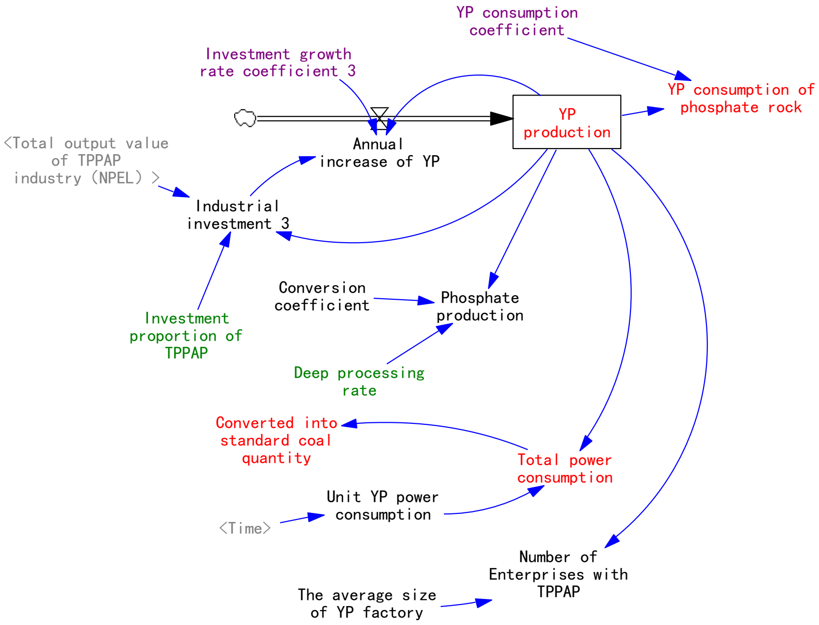

Fig. S3 SFDs of the traditional TPPAP module.

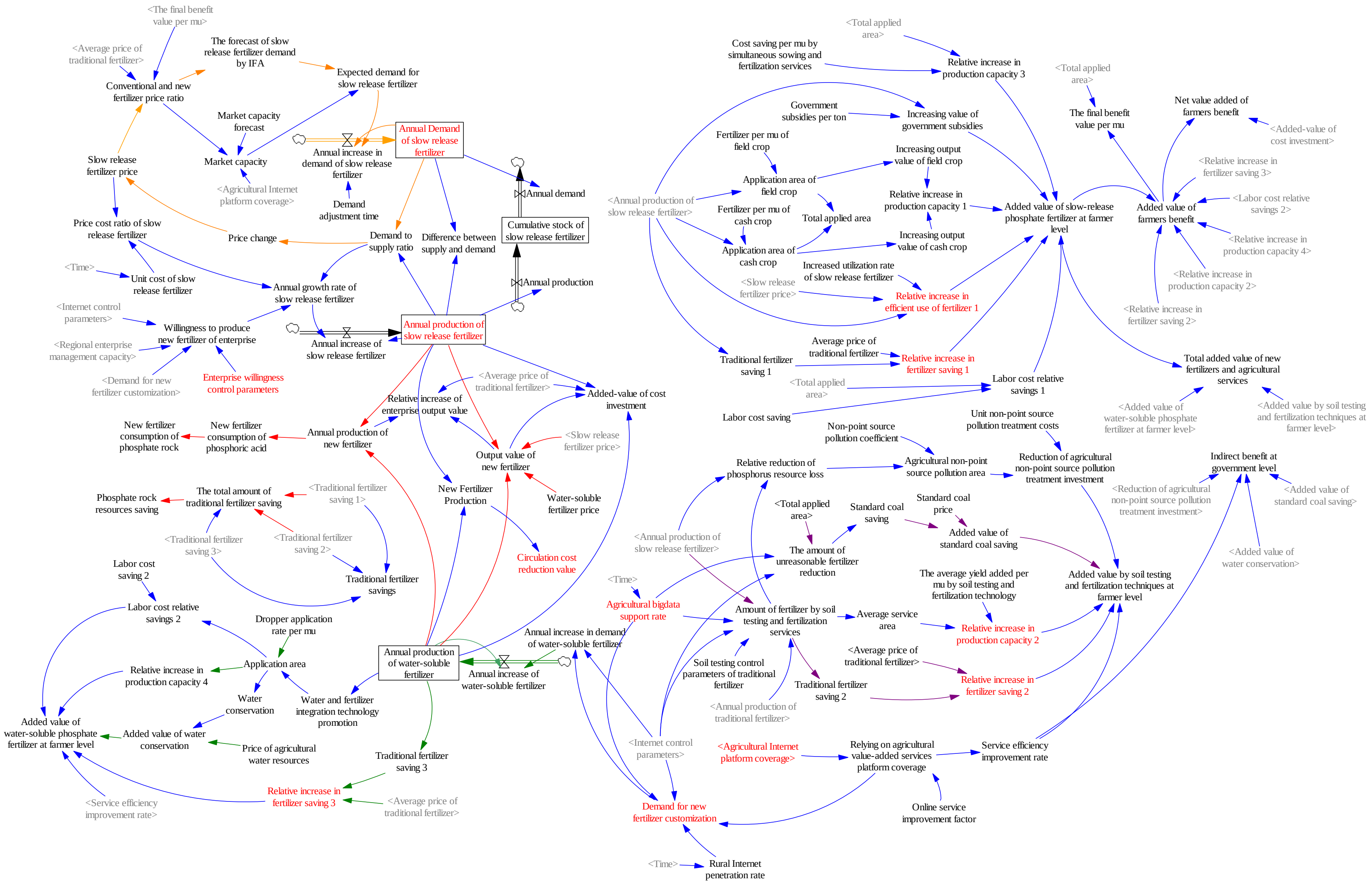

Fig.S4 The SFD of a new fertilizer module based on “Internet+” technology.

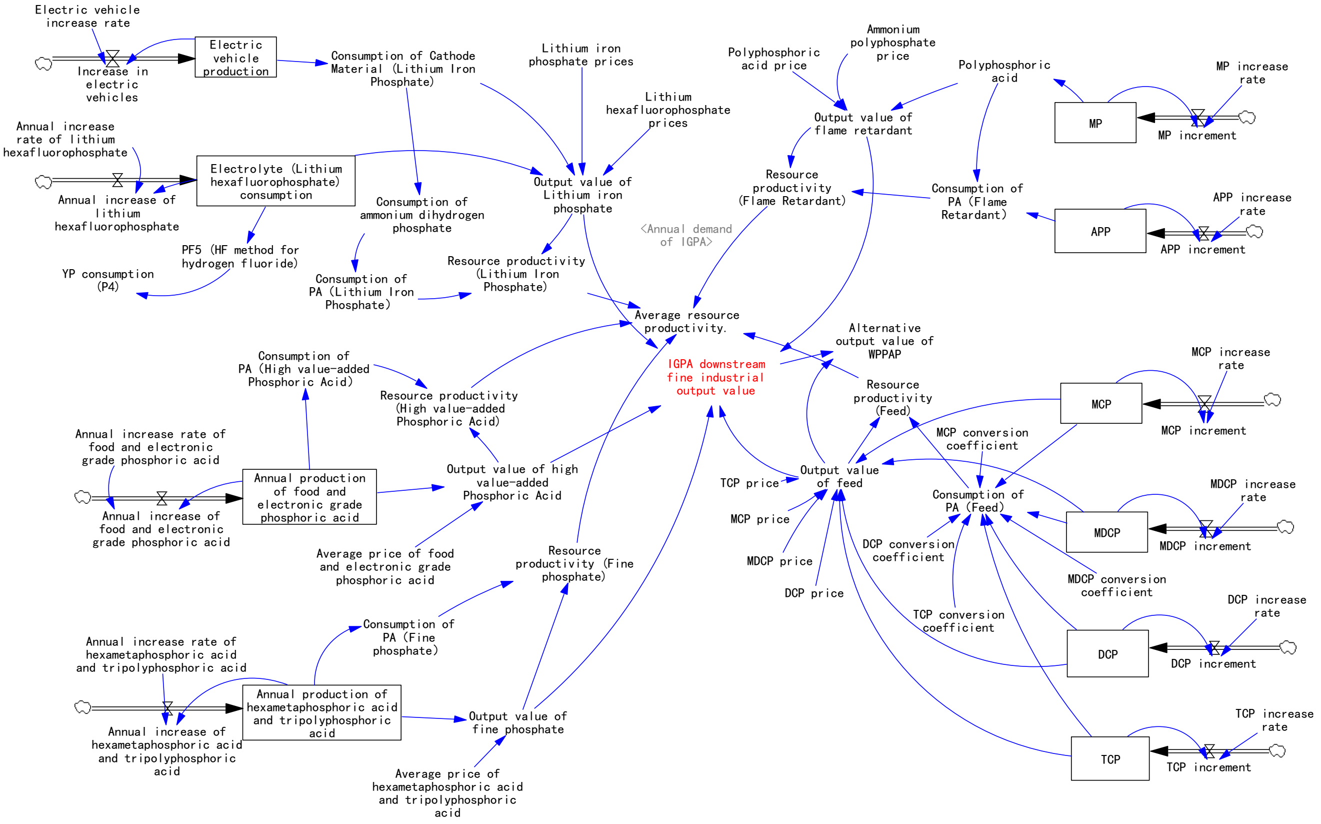

Fig. S5 SFDs of the IGPA downstream module.

1.2.3 The main variables and function relations in industrial subsystem

The industrial subsystem contains 251 variables, including 19 state variables. The equations between the main variables are shown in Table S2.

Table S2 Main variables and their equations in industrial subsystem.

Module	Variable	The type of the variable	Unit	Equation
Traditional WPPAP module	Annual production of HCPF	State variable	Ton	Annual production of HCPF = INTEG (Annual increase of HCPF, Initial value)
	Annual production of LCPF	Auxiliary variable	Ton	Annual production of LCPF =SMOOTH(Initial value 1*(1+ Annual growth rate of LCPF)^(Time- Initial year),

				2)
	Cumulative stock of PF	State variable	Ton	Cumulative stock of PF = INTEG (Expected annual production of PF - Annual Demand of PF,0)
	Demand for PF production	Auxiliary variable	Ton	Demand for PF production =MAX(0, Market demand forecast of PF +(Expected stock of PF- Cumulative stock of PF)/ Stock adjustment time)
	Market demand forecast of PF	Auxiliary variable	Ton	Market demand forecast of PF =SMOOTH(Annual Demand of PF, Moving average time)
	Accumulated stock of IGPA	State variable	Ton	Accumulated stock of IGPA = INTEG (Annual production of IGPA - Expected annual demand for IGPA, Initial value)
	Demand for IGPA production	Auxiliary variable	Ton	Demand for IGPA production =MAX(0, Market demand forecast of IGPA +(Expected stock of IGPA - Accumulated stock of IGPA)/ Stock adjustment time 2)
	Market demand forecast of IGPA	Auxiliary variable	Ton	Market demand forecast of IGPA =SMOOTH(Expected annual demand for IGPA , Move time 2)
	Annual stock of PA	State variable	Ton	Annual stock of PA = INTEG (Annual stock change,0)
Traditional TPPAP module	YP production	State variable	Ton	YP production = INTEG (Annual increase of YP, Initial value of YP production)
	Phosphate production	Auxiliary	Ton	Phosphate production = Deep processing rate * YP production * Conversion

		variable		coefficient
	Annual increase of YP	Auxiliary variable	Ton	Annual increase of YP =IF THEN ELSE(YP production <=150000, Industrial investment 3/" Investment growth rate coefficient 3" ,0)
New fertilizer module	Annual Demand of SCRF	State variable	Ton	Annual Demand of SCRF = INTEG (Annual increase in demand of SCRF,30000)
	Annual production of SCRF	State variable	Ton	Annual production of SCRF = INTEG (Annual increase of SCRF,30000)
	SCRF price	Auxiliary variable	Ton /yuan	SCRF price =3700+ Price change *1000
	Demand to supply ratio	Auxiliary variable	Dmnl	"Demand to supply ratio" = ABS(Annual Demand of SCRF / Annual production of SCRF)
	Conventional and new fertilizer price ratio	Auxiliary variable	Dmnl	Conventional and new fertilizer price ratio =IF THEN ELSE(SCRF price >= The final benefit value per mu, Average price of traditional fertilizer /(SCRF price - The final benefit value per mu) , 2)
	Annual production of water-soluble fertilizer	State variable	Ton	Annual production of water-soluble fertilizer = INTEG (Annual increase of water-soluble fertilizer, 300)
	Annual production of new fertilizer	Auxiliary variable	Ton	Annual production of new fertilizer = Annual production of water-soluble fertilizer *0.2+ Annual production of SCRF *0.15

IGPA downstream module	Consumption of Cathode Material (Lithium Iron Phosphate)	Auxiliary variable	Ton	Consumption of Cathode Material (Lithium Iron Phosphate)= Electric vehicle production *0.052
	Electrolyte (Lithium hexafluorophosphate) consumption	State variable	Ton	Electrolyte (Lithium hexafluorophosphate) consumption = INTEG (Annual increase of lithium hexafluorophosphate,1000)
	APP	State variable	Ton	APP= INTEG (APP increment,8000)
	Consumption of PA(Flame Retardant)	Auxiliary variable	Ton	Consumption of PA(Flame Retardant)= Polyphosphoric acid + APP*(98/97)
	Consumption of PA(High value-added Phosphoric Acid)	Auxiliary variable	Ton	Consumption of PA(High value-added Phosphoric Acid)=" Annual production of food and electronic grade phosphoric acid "*0.72
	Consumption of PA(Feed)	Auxiliary variable	Ton	Consumption of PA(Feed)=MCP conversion coefficient * MCP +MDCP*MDCP conversion coefficient + DCP *DCP conversion coefficient +TCP*TCP conversion coefficient

1.3 Economic Subsystem

1.3.1 Causal analysis of the economic subsystem

According to the production process of industrial subsystems, the economic subsystem includes the TPPAP and WPPAP economic subsystems. Output variables include the output value of phosphorus products, non-phosphorus products, agricultural services and environmental impacts on the economy. The economic subsystem also includes

four modules: the WPPAP economic module, the TPPAP economic module, the total economic module and the resource productivity module.

1.3.2 SFDs of the economic subsystem

The total output value of the phosphorus resource industry includes the output value of the WPPAP industry, the TPPAP industry, non-phosphorus products with WPPAP and non-phosphorus products with TPPAP. Further, we consider non-phosphorus products and economic losses, and the four types of output are classified as follows: non-phosphorus output value and economic loss (NPEL), non-phosphorus output value without economic loss (NPNEL), economic loss without non-phosphorus output value (NNPEL), and without non-phosphorus output and economic loss (NNPNEL). According to the requirements of our study, the total output value is divided into the total output value of the phosphorus resources industry (NPEL), the total output value of the phosphorus resources industry (NPNEL), the total output value of the phosphorus resources industry (NNPEL), and the total output value of the phosphorus resources industry (NNPNEL) (Fig.S6).

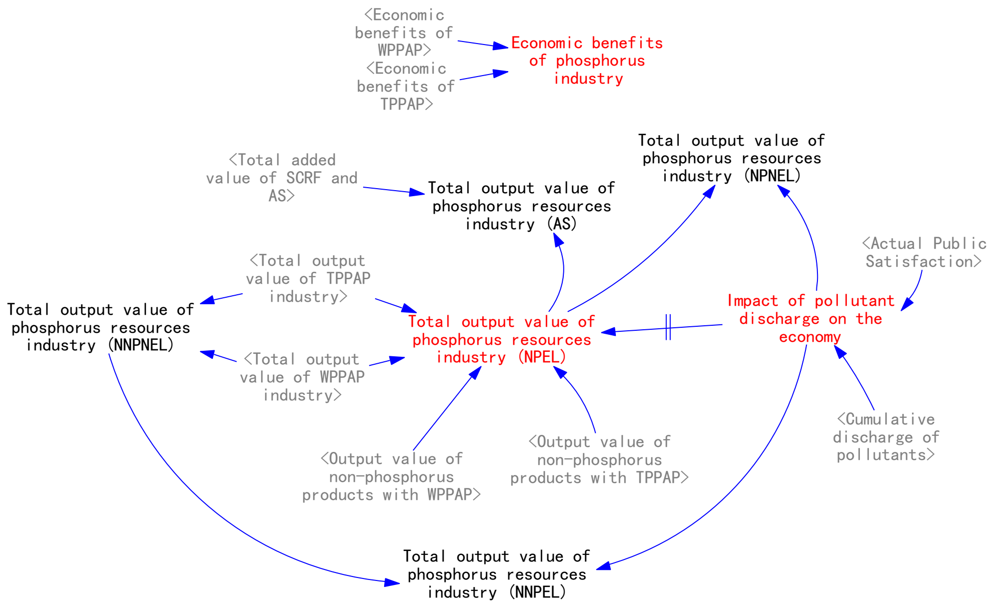

Fig. S6 SFDs of the phosphorus resources industry total economic module.

According to the WPPAP, the output value mainly includes the total output value of PF, which includes the total output value of traditional PF, new PF, and IGPA downstream industrial output (Fig.S7). When considering the output value of non-phosphorus products with WPPAP and the impact of pollutant discharge on the economy, it is necessary to set the total output value of the WPPAP industry (NPEL).

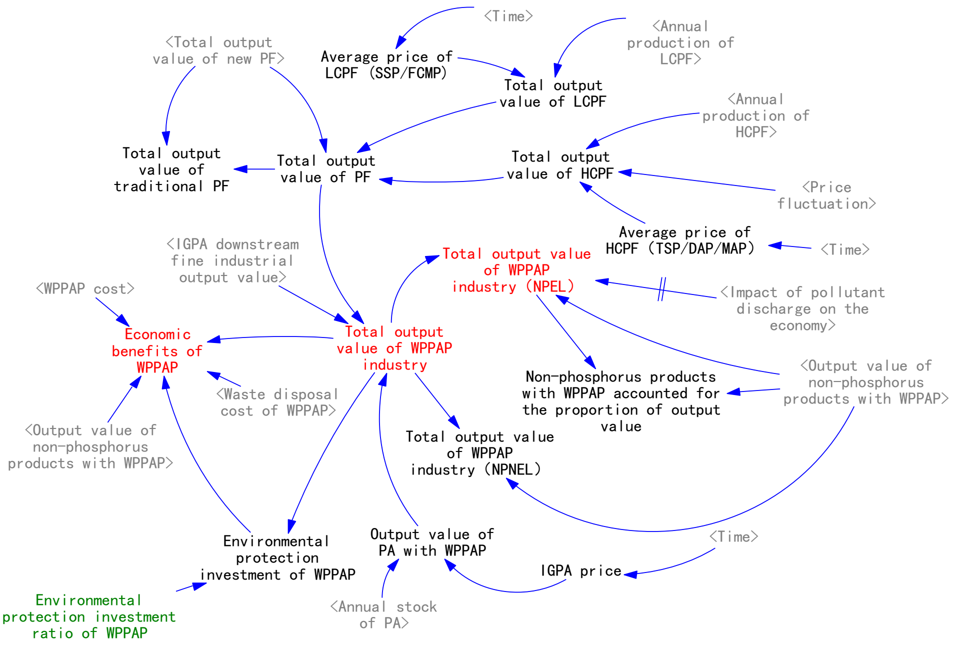

Fig. S7 SFDs of the WPPAP economic module.

The output value of the TPPAP industry includes the output value of YP and phosphate (Fig.S8). Considering the output value of non-phosphorus products with TPPAP and the impact of pollutant discharge on the economy, set the total output value of the TPPAP industry (NPEL).

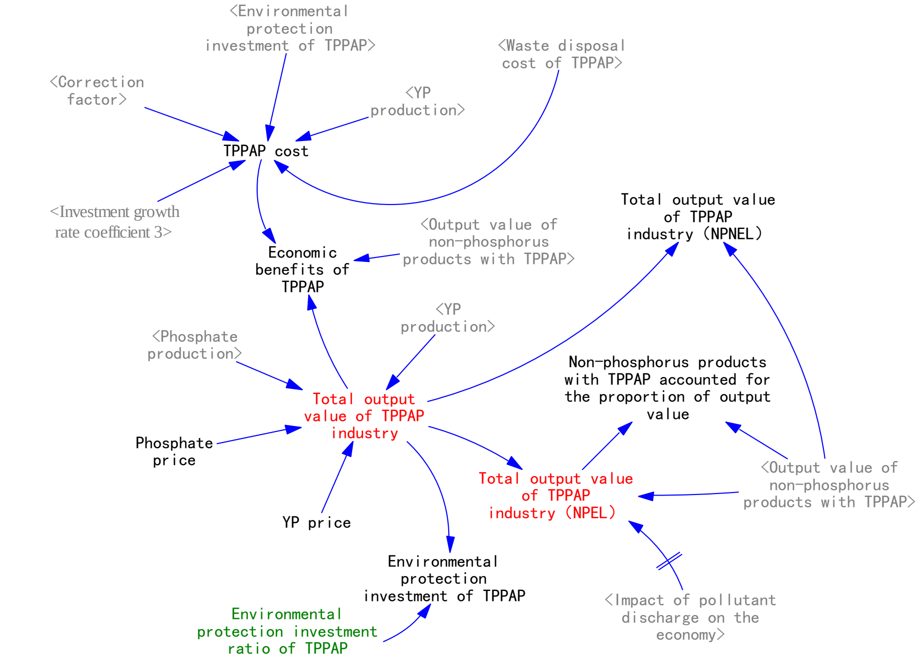

Fig. S8 SFDs of the TPPAP economic module.

The resource productivity [13] of phosphorus resources is calculated as follows: Resource Productivity = total output value of phosphorus resources / PR consumption. The rate of resource output takes into account resource productivity, resource productivity (AS), resource productivity (NNP), and the resource productivity of WPPAP and

TPPAP. The calculation of different resource yield rates is based on their physical meaning, and numerator and denominator values are different (Fig.S9).

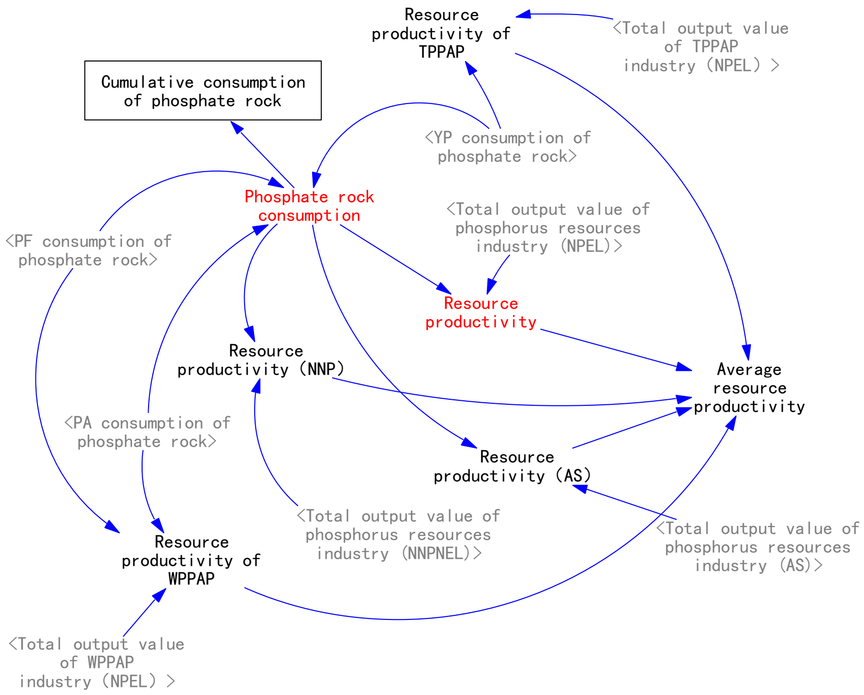

Fig. S9 SFDs of the resource productivity module.

1.3.3 The main variables and function relations in economic subsystem

A total of 72 variables are included in the economic subsystem. The equations for the major variables are shown in Table S3.

Table S3 Main variables and their equations in economic subsystem.

Module	Variable	The type of the variable	Unit	Equation
Phosphorus resources industry total economic module	Total output value of phosphorus resources industry	State variable	Yuan	Total output value of phosphorus resources industry (NPEL)= Total output value of TPPAP industry + Total output value of WPPAP industry + Output value of non-phosphorus products with WPPAP + Output value

	(NPEL)			of non-phosphorus products with TPPAP -1.5*DELAY1(Impact of pollutant discharge on the economy, 2)
	Total output value of phosphorus resources industry (NPNEL)	State variable	Yuan	Total output value of phosphorus resources industry (NPNEL)= Total output value of phosphorus resources industry (NPEL) +1.5*DELAY1(Impact of pollutant discharge on the economy, 2)
	Total output value of phosphorus resources industry (NNPEL)	State variable	Yuan	Total output value of phosphorus resources industry (NNPEL)= Total output value of phosphorus resources industry (NNPNEL)- 1.5*DELAY1(Impact of pollutant discharge on the economy, 2)
	Total output value of phosphorus resources industry (NNPNEL)	State variable	Yuan	Total output value of phosphorus resources industry (NNPNEL)= Total output value of WPPAP industry + Total output value of TPPAP industry
	Total output value of PF	State variable	Yuan	Total output value of PF = Total output value of LCPF + Total output value of HCPF + Total output value of new PF
WPPAP economic	Total output value of	State variable	Yuan	Total output value of WPPAP industry = Output value of PA with WPPAP +

module	WPPAP industry			Total output value of PF + IGPA downstream fine industrial output value
	Total output value of WPPAP industry(NPEL)	State variable	Yuan	Total output value of WPPAP industry(NPEL)= Total output value of WPPAP industry + Output value of non-phosphorus products with WPPAP -DELAY1(Impact of pollutant discharge on the economy, 2)
	Total output value of WPPAP industry(NPNEL)	State variable	Yuan	Total output value of WPPAP industry(NPNEL)= Total output value of WPPAP industry + Output value of non-phosphorus products with WPPAP
	Output value of non-phosphorus products with WPPAP	State variable	Yuan	Output value of non-phosphorus products with WPPAP =" Annual output value of comprehensive utilization of waste gas containing fluorine and iodine "+ Annual output value of PG
	Total output value of new PF	State variable	Yuan	Total output value of new PF = Water-soluble fertilizer price * Annual production of water-soluble fertilizer + SCRF price * Annual production of SCRF
	Total added value of new	State variable	Yuan	Total added value of new fertilizers and AS = Total added value of SCRF

	fertilizers and AS			and AS + Added value of water-soluble fertilizer
	IGPA downstream fine industrial output value	State variable	Yuan	IGPA downstream fine industrial output value =Output value of Lithium iron phosphate + Output value of high value-added Phosphoric Acid + Output value of flame retardant + Output value of feed + Output value of fine phosphate
TPPAP economic module	Output value of non-phosphorus products with TPPAP	State variable	Yuan	Output value of non-phosphorus products with TPPAP = Formic acid production * Formic acid price + Fine powder production * Fine powder price
	Total output value of TPPAP industry	State variable	Yuan	Total output value of TPPAP industry = YP production *YP price*0.3+ Phosphate production * Phosphate price
	Total output value of TPPAP industry(NPNEL)	State variable	Yuan	Total output value of TPPAP industry(NPNEL)= Total output value of TPPAP industry + Output value of non-phosphorus products with TPPAP
Resource productivity module	Resource productivity	State variable	Yuan/ ton	Resource productivity = Total output value of phosphorus resources industry (NPEL)/ PR consumption
	Resource	State	Yuan/	Resource productivity(AS)= Total

productivity(AS)	variable	ton	output value of phosphorus resources industry (AS)/ PR consumption
Resource productivity(NNP)	State variable	Yuan/ ton	Resource productivity(NNP)= Total output value of phosphorus resources industry (NNPNEL)/ PR consumption

1.4 Environmental subsystem

1.4.1 Causal analysis of the environmental subsystem

The environmental subsystem is dependent on the industrial subsystem. In the process of producing phosphorus products, phosphorus-containing and non-phosphorus pollutants are generated, and pollutants discharged into the ecological environment produce environmental pollution, cause air pollution, groundwater damage and land use issues, and result in resource waste to a certain degree. Therefore, it is necessary to pay attention to waste recycling, safe disposal methods and other issues throughout economic development. The environmental subsystem presented in this paper covers the entire process of waste generation, accumulation and reutilization. The feedback relationship between the environmental subsystem and the economic and the social subsystem is discussed based on the effects of pollutant discharge on the economy and society.

1.4.2 SFDs of the environmental subsystem

WPPAP and its downstream environmental modules include the production, storage and treatment of phosphogypsum (PG) [14] and fluorine-containing waste gas (Fig.S10). PG and fluorine-containing waste gas discharge standards must consider safe handling and comprehensive utilization, and other emissions will require sewage charges. The comprehensive utilization of PG is mainly applied for the production of phosphogypsum brick, plaster board, cement release agents and other products [15, 16]. Fluorine and iodine exhaust gas utilization involves the production of hydrogen fluoride, ammonium hydrogen fluoride, iodine products and so on [17].

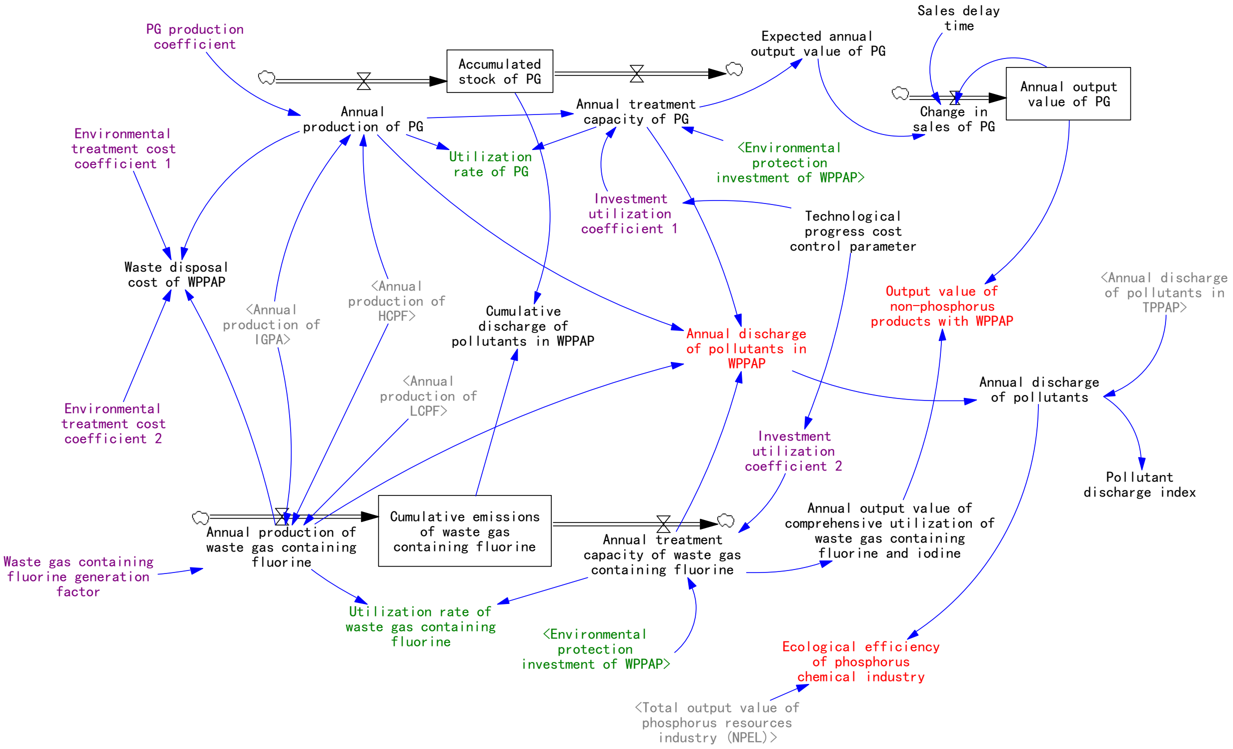

Fig. S10 SFDs of the WPPAP environmental module.

We consider the comprehensive utilization and environmental treatment of pollutants downstream of TPPAP, including the safe disposal and comprehensive utilization of phosphorus slag, phosphate mud, ferrophosphorus and YP tail gas [18] (Fig.S11). Synthesizing TPPAP solid waste involves producing a fine powder, and YP tail gas synthesizing involves producing formic acid [19].

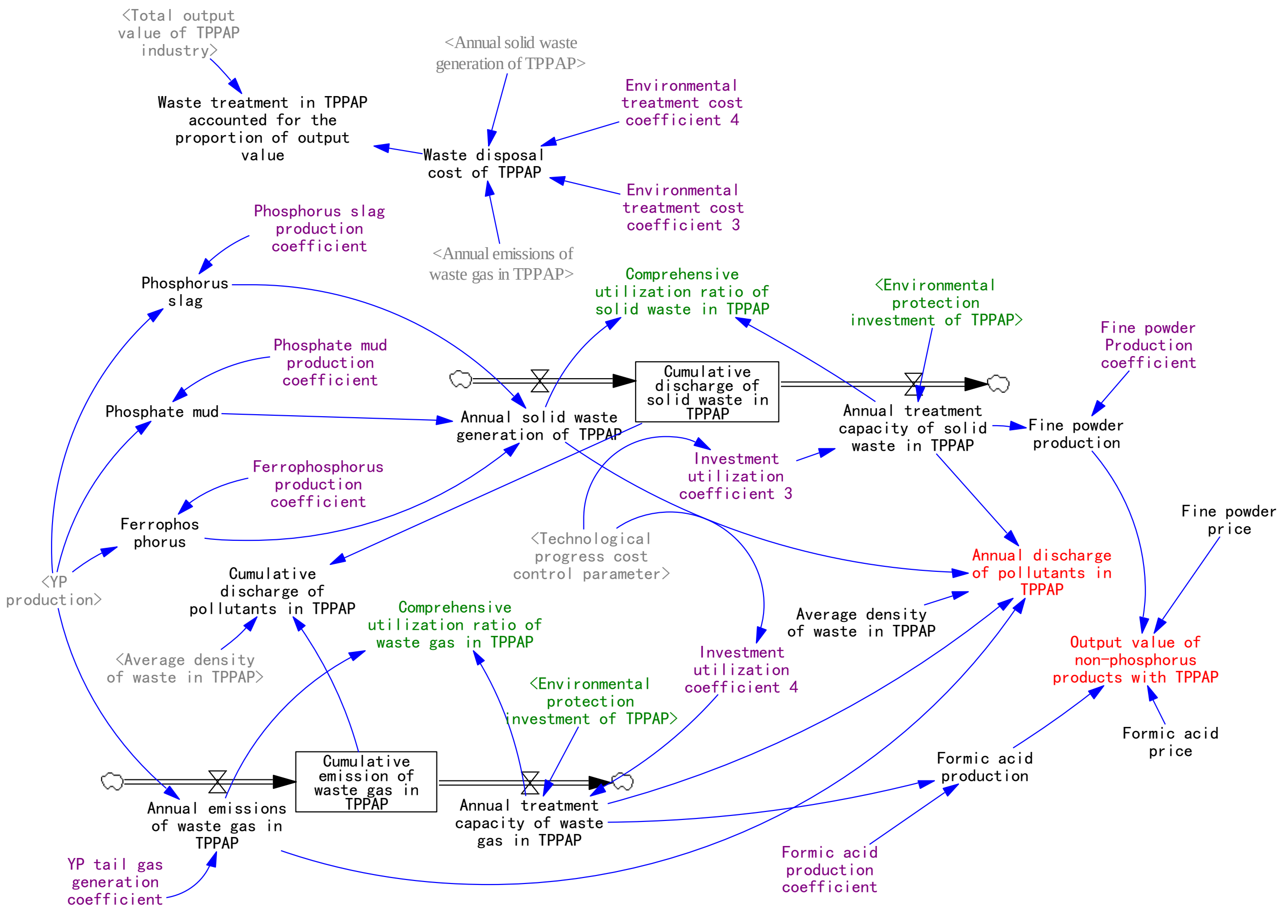

Fig. S11 SFDs of the TPPAP environmental module.

1.4.3 The main variables and function relations in environmental subsystem

The environmental subsystem contains 60 variables, including 19 state variables. The equations for the major variables are shown in Table S4.

Table S4 Main variables and their equations in environmental subsystem.

Module	Variable	The type of the variable	Unit	Equation
WPPAP environmental module	Annual production of PG	Auxiliary variable	Ton	Annual production of PG = PG production coefficient *(Annual production of HCPF + Annual production of IGPA)

Accumulated stock of PG	State variable	Ton	Accumulated stock of PG = INTEG (Annual production of PG - Annual treatment capacity of PG, Initial value)
Annual treatment capacity of PG	Auxiliary variable	Ton	Annual treatment capacity of PG =0.5* Environmental protection investment of WPPAP /" Investment utilization coefficient 1"
Utilization rate of PG	Auxiliary variable	Dmnl	Utilization rate of PG =IF THEN ELSE(Annual treatment capacity of PG / Annual production of PG <=1, Annual treatment capacity of PG / Annual production of PG, 1)
Annual production of waste gas containing fluorine	Auxiliary variable	Ton	Annual production of waste gas containing fluorine = (Annual production of LCPF + Annual production of HCPF + Annual production of IGPA)* Waste gas containing fluorine generation factor
Cumulative emissions of waste gas containing fluorine	State variable	Ton	Cumulative emissions of waste gas containing fluorine = INTEG (Annual production of waste gas containing fluorine - Annual treatment capacity of waste gas containing fluorine, Emission initial value)
Annual treatment capacity of waste gas	Auxiliary variable	Ton	Annual treatment capacity of waste gas containing fluorine =0.5* Environmental protection investment of WPPAP /"Investment utilization

	containing fluorine			coefficient 2"
	Utilization rate of waste gas containing fluorine	Auxiliary variable	Dmnl	Utilization rate of waste gas containing fluorine =IF THEN ELSE(Annual treatment capacity of waste gas containing fluorine / Annual production of waste gas containing fluorine <=1, Annual treatment capacity of waste gas containing fluorine / Annual production of waste gas containing fluorine, 1)
	Cumulative discharge of pollutants	State variable	Ton	Cumulative discharge of pollutants = Annual discharge of pollutants in WPPAP + Annual discharge of pollutants in TPPAP
	Pollutant discharge index	Auxiliary variable	Dmnl	Pollutant discharge index = (LN(Cumulative discharge of pollutants)-16)*5
TPPAP environmental module	Annual solid waste generation of TPPAP	Auxiliary variable	Ton	Annual solid waste generation of TPPAP = Phosphorus slag + Phosphate mud + Ferrophosphorus
	Cumulative discharge of solid waste in TPPAP	State variable	Ton	Cumulative discharge of solid waste in TPPAP =INTEG (Annual solid waste generation of TPPAP - Annual treatment capacity of solid waste in TPPAP,1.411e+007)
	Annual	Auxiliary	Ton	Annual treatment capacity of solid

treatment capacity of solid waste in TPPAP	variable		waste in TPPAP = 0.5* Environmental protection investment of TPPAP /"Investment utilization coefficient 3"
Comprehensive utilization ratio of solid waste in TPPAP	Auxiliary variable	Dmnl	Comprehensive utilization ratio of solid waste in TPPAP =IF THEN ELSE(Annual treatment capacity of solid waste in TPPAP / Annual solid waste generation of TPPAP <=1, Annual treatment capacity of solid waste in TPPAP/ Annual solid waste generation of TPPAP, 1)
Annual emissions of waste gas in TPPAP	Auxiliary variable	Ton	Annual emissions of waste gas in TPPAP = YP tail gas generation coefficient * YP production
Annual treatment capacity of waste gas in TPPAP	Auxiliary variable	Ton	Annual treatment capacity of waste gas in TPPAP =0.5* Environmental protection investment of TPPAP /" Investment utilization coefficient 4"
Comprehensive utilization ratio of waste gas in TPPAP	Auxiliary variable	Dmnl	Comprehensive utilization ratio of waste gas in TPPAP =IF THEN ELSE(Annual treatment capacity of waste gas in TPPAP / Annual emissions of waste gas in TPPAP <=1, Annual treatment capacity of waste gas in TPPAP / Annual emissions of waste gas

in TPPAP, 1)

1.5 Social subsystem

1.5.1 Causal analysis of the social subsystem

In the social subsystem, the government, as the macroscopic main body of the industrial cluster, plays a major role in creating the innovation environment [20]. It mainly directs policy-making, financial capital support, technical assistance and market protection [21]. In this study, government support of the social innovation system is mainly reflected through financial expenditures (e.g., investments in universities and research institutes, investments in support service system construction, investments in education and training and investments in innovative culture construction). Direct investments in universities and scientific research units constitute the most effective means to enhance the productivity of science and technology, which is conducive to the efficient combination of industry, the academy, research and politics and to the smooth transformation of scientific research achievements. For enterprises and industrial strategy consulting firms, technology incubation centers and support from scientific and technological intermediaries will directly affect scientific research achievements throughout industrialization and the quality of science and technology services. Investments in regional education and talent training can increase the number of technical service personnel employed in the phosphorus resources industry. As the main force behind scientific and technological progress and as an important carrier of innovative knowledge, the quality of scientific research personnel directly affects the innovation quality of the industrial cluster. The above three forms constitute the most common modes of local government support and the most direct ways to promote innovation. However, an emphasis on innovation culture and on the construction of service systems can ensure the long-term enhancement of regional innovation capacities. The promotion of innovative cultures and the creation of innovative atmospheres are conducive to raising awareness of innovation in society as a whole. Supporting the construction of supporting service systems is conducive to providing hardware protection across society, thus enhancing the overall technological service capabilities of an industry. Therefore, we present relevant variables to describe scientific and technological services that enhance capacities and atmospheres of innovation for regional innovation and the causal role of revenues.

In the social innovation subsystem, an increase in knowledge stock will strengthen regional innovation [22] and further improve regional technologies, thus increasing the regional product market share while reducing market competition pressure. Awareness of innovation and willingness on the part of regional enterprises are important variables that reflect enterprise participation in innovation activities. These relevant variables in different ways work

together to support awareness of and enthusiasm for enterprise innovation. The number of variables involved in the causal cycle of the system dynamics model is as high as 373. Therefore, we present a relevant feedback loop to examine regional enterprise innovation awareness of regional innovation income causality.

Naturally, higher levels of pollutant emissions are bound to result in public dissatisfaction, and so the relationship between public satisfaction and the pollutant discharge index is linked to industrial and social systems[23, 24].

1.5.2 SFDs of the social subsystem

The impact of pollutant discharge on public satisfaction involves a delay period expressed as a time variable of adjustment (Fig.S12).

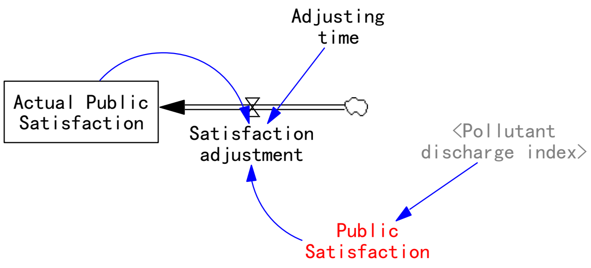

Fig. S12 SFDs of the impact of pollutant discharge on public satisfaction.

The policy constraint index is influenced by annual pollutant discharge levels and the policy support index is influenced by regional innovation capacities. The two act as policy support and constraint auxiliary variables, thus controlling financial expenditure effects on innovation [25] (Fig.S13). Financial expenditures through different support channels and investment intensity levels affect the use of union funds and the construction of soft power in regional science and technology innovation. Enterprise decision making in the social system results from a change in policy support and also affects the construction of regional technological innovation capacities.

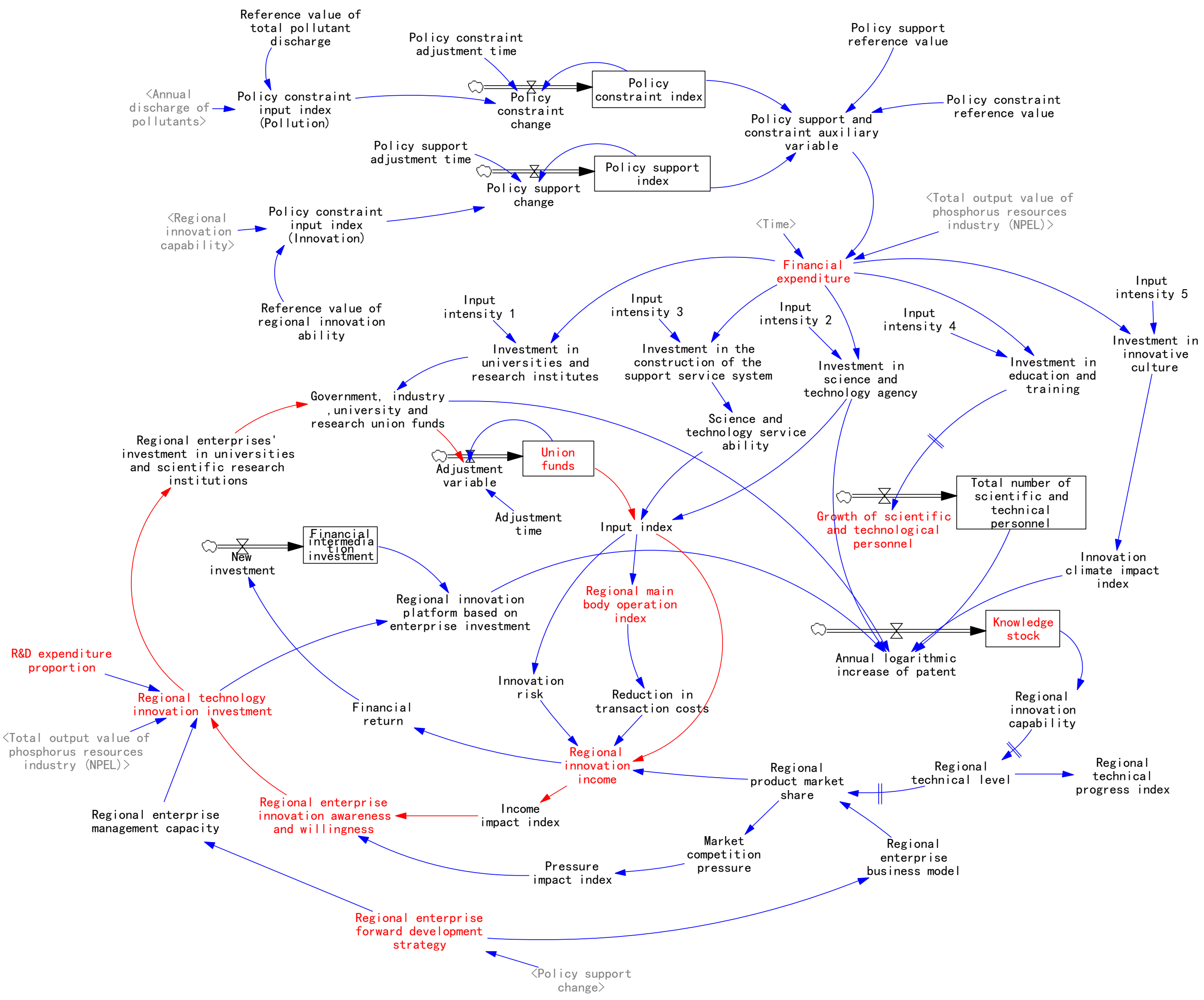

Fig. S13 SFDs of the social subsystem.

1.5.3 The main variables and function relations in social subsystem

The social subsystem includes 63 variables, including 6 state variables. Equations for the main variables are shown in Table S5.

Table S5 Main variables and their equations in social subsystem.

Variable	The type of the variable	Unit	Equation
Public Satisfaction	State variable	Dmnl	Public Satisfaction = 5- Pollutant discharge index

Policy constraint input index (Pollution)	Auxiliary variable	Ton/ton	Policy constraint input index (Pollution)= Annual discharge of pollutants / Reference value of total pollutant discharge
Policy constraint input index (Innovation)	Auxiliary variable	Dmnl	Policy constraint input index (Innovation)= Regional innovation capability / Reference value of regional innovation ability
Policy constraint index	State variable	Dmnl	Policy constraint index = INTEG (Policy constraint change,1)
Policy support index	State variable	Dmnl	Policy support index = INTEG (Policy support change,1)
Policy support and constraint auxiliary variable	Auxiliary variable	Dmnl	Policy support and constraint auxiliary variable = Policy constraint index / Policy constraint reference value - Policy support index / Policy support reference value
Financial expenditure	Auxiliary variable	Yuan	Financial expenditure =IF THEN ELSE(Time=2014, Total output value of phosphorus resources industry (NPEL)*0.17, DELAY1(Total output value of phosphorus resources industry (NPEL))*0.17*(1+ Policy support and constraint auxiliary variable) , 2))
Union funds	State variable	Dmnl	Union funds = INTEG (Adjustment variable,0)
Knowledge stock	State variable	Dmnl	Knowledge stock = INTEG (Annual logarithmic increase of patent,600)

Innovation climate impact index	Auxiliary variable	Dmnl	Innovation climate impact index = 0.068*LN(ABS(Investment in innovative culture))+0.34
Input index	Auxiliary variable	Dmnl	Input index =(Union funds + Investment in science and technology agency)* Science and technology service ability
Regional technical level	Auxiliary variable	Dmnl	Regional technical level =DELAY1(Regional innovation capability , 2)
Regional innovation income	Auxiliary variable	Yuan	Regional innovation income = DELAY1(Input index *SMOOTH(" Regional product market share " , 2)-SMOOTH(Innovation risk, 2)-Reduction in transaction costs, 2)
Regional technology innovation investment	Auxiliary variable	Yuan	Regional technology innovation investment = MAX(Total output value of phosphorus resources industry (NPEL)* Regional enterprise management capacity * Regional enterprise innovation awareness and willingness *"R&D expenditure proportion", 0)
Regional innovation platform based on enterprise investment	Auxiliary variable	Yuan	Regional innovation platform based on enterprise investment = Financial intermediation investment +0.5* Regional technology innovation investment
Regional enterprise innovation awareness and	Auxiliary variable	Dmnl	Regional enterprise innovation awareness and willingness =IF THEN ELSE((Income impact index - Pressure impact index)>0, Income impact index - Pressure impact index, 0)

willingness			
Regional enterprise forward development strategy	Auxiliary variable	Dmnl	Regional enterprise forward development strategy =DELAY1(Policy support change +0.5, 1)

2 Data source

The main sources of historical data of each subsystem and relevant technical parameters are shown in the tables below.

Table S6 The historical data of the main parameters of the resource subsystem.

Year	2005	2006	2007	2008	2009	2010	2011	2012	2013
Annual amount of PR mining (Unit: Mt)	8.79	10.11	11.57	13.19	13.98	15.6	20.28	23.73	27.29
Annual ore dressing capacity (Unit: Mt)	7.03	8.09	9.26	10.55	11.19	12.48	16.23	18.98	21.83
PR output (Unit: Mt)	0.85	1.23	1.70	2.09	1.17	3.45	6.94	9.04	11.13
Annual increase in PR (Unit: Mt)	77.46	66.67	57.39	49.39	42.51	36.59	31.49	27.11	23.33
Resources and environment carrying capacity (Unit: Mt)	700.00	758.26	804.59	839.92	865.05	882.04	890.36	885.59	869.92

Table S7 The historical data of the main parameters of the industrial subsystem.

Year	2005	2006	2007	2008	2009	2010	2011	2012	2013

Annual production of HCPF (Unit: Mt)	1.06	1.16	1.25	1.35	1.53	1.74	1.90	2.16	2.41
Annual production of LCPF (Unit: Mt)	0.30	0.27	0.24	0.22	0.20	0.18	0.16	0.14	0.13
Annual production of IGPA (Unit: ton)	0.0001	27,395	54,693	108,202	168,504	215,866	288,521	360,664	438,678
PA production (Unit: ton)	150,000	182,990	218,482	262,653	338,377	175,172	131,408	97,867	71,819
PF consumption of PR (Unit: Mt)	4.53	4.75	4.98	5.21	5.76	6.39	6.88	7.66	8.45
PA consumption of PR (Unit: Mt)	0.00033	0.091	0.18	0.36	0.56	0.72	0.96	1.20	1.46
YP consumption of PR (Unit: Mt)	1.65	2.01	2.40	2.89	3.72	1.93	1.45	1.08	7.90
PR consumption (Unit: Mt)	6.18	6.85	7.56	8.46	10.04	9.03	9.28	9.94	10.7
Cumulative consumption of PR (Unit: Mt)	0	6.18	13.04	20.6	29.06	39.10	48.13	57.42	67.36

Table S8 The historical data of the main parameters of the economic subsystem.

Year	2005	2006	2007	2008	2009	2010	2011	2012	2013
Total output value of WPPAP industry (Unit: Billion yuan)	5.81	5.79	11.36	12.81	10.08	15.46	15.38	16.66	17.32
Output value of non-phosphorus products with	122.09	121.66	238.61	269.09	211.69	324.76	322.97	349.84	363.63

WPPAP (Unit: Million yuan)									
Total output value of WPPAP industry(NPEL)(Unit: Billion yuan)	5.94	5.92	11.59	13.07	10.26	15.74	15.63	16.9	17.54
Total output value of WPPAP industry(NPNEL)(Unit: Billion yuan)	5.94	5.92	11.60	13.08	10.29	15.79	15.70	17.01	17.68
Economic benefits of WPPAP (NEL)(Unit: Billion yuan)	2.58	2.15	7.37	8.30	4.68	9.27	8.33	8.52	8.05
Total output value of TPPAP industry (Unit: Billion yuan)	1.14	1.23	1.53	2.63	2.48	1.39	1.08	0.86	0.62
Output value of non-phosphorus products with TPPAP (Unit: Million yuan)	42.19	45.39	56.62	97.19	91.53	52.54	40.68	32.14	22.66
Total output value of TPPAP industry(NPEL)(Unit: Billion yuan)	1.19	1.28	1.59	2.72	2.55	1.42	1.09	0.84	0.57
Total output value of TPPAP industry(NPNEL)(Unit:	1.19	1.28	1.59	2.73	2.57	1.44	1.12	0.90	0.65

Billion yuan)									
Economic benefits of TPPAP (NEL)(Unit: Million yuan)	44.33	-114.91	-69.94	724.46	-4.16	105.70	119.74	149.43	98.61
Total output value of phosphorus resources industry (NPEL) (Unit: Billion yuan)	7.12	7.19	13.18	15.79	12.82	17.16	16.72	17.75	18.11
Total output value of phosphorus resources industry (NPNEL) (Unit: Billion yuan)	7.12	7.19	13.19	15.81	12.86	17.23	16.82	17.90	18.32
Total output value of phosphorus resources industry (NNPNEL) (Unit: Billion yuan)	6.96	7.02	12.90	15.45	12.56	16.85	16.46	17.52	17.94
Total output value of phosphorus resources industry (NNPEL) (Unit: Billion yuan)	6.956	7.02	12.89	15.42	12.51	16.78	16.35	17.36	17.73
Total output value of phosphorus resources industry (AS) (Unit: Billion yuan)	7.12	7.19	13.18	15.78	12.8	17.13	16.68	17.69	18.04
Economic benefits of phosphorus industry (Unit: Billion yuan)	2.63	2.03	7.30	9.02	4.67	9.37	8.45	8.67	8.15

Table S9. The historical data of the main parameters of the environmental subsystem.

Year	2005	2006	2007	2008	2009	2010	2011	2012	2013
Cumulative discharge of pollutants in WPPAP (Unit: Mt)	0	5.16	10.93	17.14	24.04	32.25	41.57	52.07	64.15
Annual discharge of pollutants in WPPAP (Unit: Mt)	5.16	5.77	6.21	6.91	8.20	9.32	10.50	12.08	13.69
Cumulative discharge of pollutants in TPPAP (Unit: Mt)	0.0010	1.82	4.09	6.78	9.81	13.95	16.06	17.64	18.80
Annual discharge of pollutants in TPPAP (Unit: Mt)	1.82	2.26	2.69	3.03	4.14	2.12	1.58	1.16	0.85
Cumulative discharge of pollutants (Unit: Mt)	0.0010	6.99	15.02	23.91	33.85	46.19	57.63	69.71	82.95
Annual discharge of pollutants (Unit: Mt)	6.98	8.03	8.90	9.94	12.34	11.44	12.08	13.24	14.54
Impact of pollutant discharge on the economy (Unit: Mt)	0.002	13.97	26.8	43.06	64.2	95.11	140.3	177.72	224.56
Annual production of PG (Unit: Mt)	5.109	5.699	6.288	7.002	8.187	9.423	10.56	12.13	13.71
Annual treatment capacity of PG (Unit: ton)	145,337	144,825	284,048	320,332	251,998	386,607	384,480	416,468	432,879

Accumulated stock of PG (Unit: Mt)	0	4.964	10.52	16.52	23.2	31.14	40.18	50.36	62.07

Table S10 The historical data of the main parameters of the social subsystem.

Year	2005	2006	2007	2008	2009	2010	2011	2012	2013
Public satisfaction	3.10	2.75	2.50	2.22	1.68	1.87	1.73	1.50	1.27
Actual public satisfaction	2.50	3.10	2.75	2.50	2.22	1.68	1.87	1.73	1.50

Table S11 The historical data of the main parameters of comprehensive indicators.

Year	2005	2006	2007	2008	2009	2010	2011	2012	2013
Ecological efficiency of phosphorus chemical industry (Unit: Yuan/ton)	1,020	895	1,481	1,587	1,037	1,498	1,381	1,337	1,240
Resource productivity (NPEL) (Unit: Yuan/ton)	1,152	1,049	1,743	1,865	1,275	1,897	1,797	1,780	1,686
Resource productivity (AS) (Unit: Yuan/ton)	1,152	1,049	1,743	1,865	1,275	1,897	1,797	1,780	1,686
Resource productivity (NNP) (Unit: Yuan/ton)	1,125	1,025	1,705	1,825	1,251	1,865	1,773	1,763	1,676
Resource productivity of TPPAP (NPEL) (Unit: Yuan/ton)	718.30	633.46	660.29	941.61	685.99	734.36	750.27	782.32	727.59
Resource productivity of WPPAP (NPEL) (Unit: Yuan/ton)	1,309	1,222	2,247	2,345	1,624	2,215	1,994	1,907	1,770

Table S12 The main relevant technical parameters in SD model.

Factor	Unit	Value#
Ore recovery rate	-	0.8
Ore dressing recovery percentage	-	0.8
Tailings utilization rate	-	0.5
PF consumption coefficient	Ton/Ton	0.3
PA consumption coefficient	Ton/Ton	0.3
YP consumption coefficient	Ton/Ton	0.3
PG production coefficient	Ton/Ton	4.82
Waste gas containing fluorine generation factor	Ton/Ton	0.16
Phosphorus slag production coefficient	Ton/Ton	10
Phosphate mud production coefficient	Ton/Ton	0.5
Ferrophosphorus production coefficient	Ton/Ton	0.1
YP tail gas generation coefficient	m^3	3000
Investment proportion of PF industry	-	0.034
PA industry investment ratio	-	0.018
YP industry investment ratio	-	0.050
Environmental protection investment ratio of WPPAP	-	0.009
Environmental protection investment ratio of TPPAP	-	0.009
Investment growth rate coefficient 1	Yuan/ton	2500
Investment growth rate coefficient 2	Yuan/ton	3875
Investment growth rate coefficient 3	Yuan/ton	11500
Investment utilization coefficient 1	Yuan/ton	180

Investment utilization coefficient 2	Yuan/ton	1282
Investment utilization coefficient 3	Yuan/ton	18.50
Investment utilization coefficient 4	Yuan/m^3	0.82
Environmental treatment cost coefficient 1	Yuan/ton	2.50
Environmental treatment cost coefficient 2	Yuan/ton	330
Environmental treatment cost coefficient 3	Yuan/ton	0.90
Environmental treatment cost coefficient 4	Yuan/ton	0.47

#Coefficient, ratio and cost are based on the specific phosphorus industry project in Wengfu Group estimates.

3 Model validation and sensitivity analysis

3.1 Model validation

According to characteristics of the SD model of industrial systems of phosphorus resources, we select variables with complete historical data for 2005 to 2013 as our object of inspection to investigate the consistency of simulation results and historical data. In the model validation, we used two variables, PR production and YP production, to compare with historical data. The simulation results show that the relative error of PR production ranges at ± 4.00% (Fig.S14a) and that the value for YP production falls between -5.00% and -1.00% (Fig.S14b). This means that the historical and simulated values of the model variables are basically the same and that the relative error is small enough and thus meets prediction requirements and can be used for subsequent analyses. Therefore, the model is considered valid.

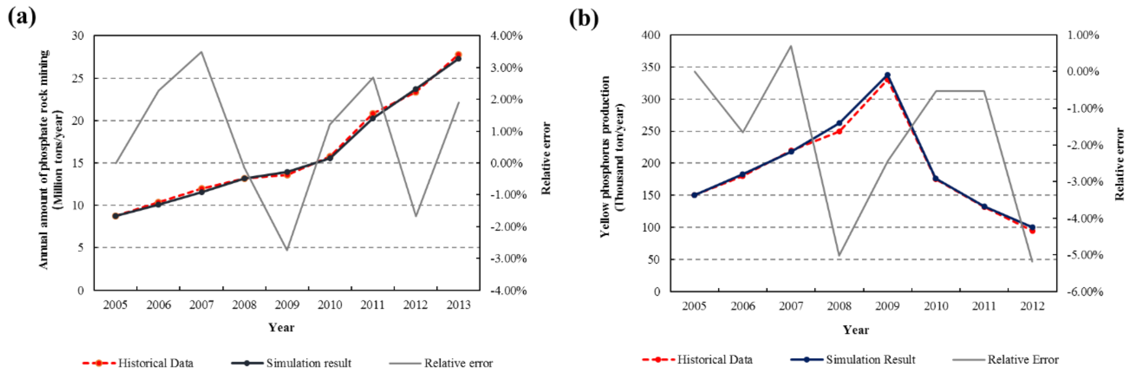

Fig. S14 Model validation. (a) The history data and simulation result comparison of annual amount of PR mining; (b) The history data and simulation result comparison of annual YP production.

3.2 Sensitivity analysis

Sensitivity analysis is designed to determine the impact of selected variables on the operational structure of the model by change of important variables of a model within a certain range and analysis of changes in model output variables [26]. Here, environmental protection investment ratio of WPPAP and investment utilization coefficient are chosen to conduct sensitivity analysis.

As is shown in Figure S15, the total output value of the WPPAP industry and the total output value of the phosphorus resources industry after 2015 should change slightly with changes in the environmental proportion. This can be attributed to the fact that the proportion of environmental change should directly affect the output value of non-phosphorus products, thereby affecting the total output value of the WPPAP industry and the total output value of the phosphorus resources industry. However, the output value of non-phosphorus products from the proportion of the total output value is small, and so the sensitivity analysis does not show a considerable response which is in accordance with the expected results of the model mechanism.

Similarly, the sensitivity analysis of investment utilization coefficient also conforms to the expected model mechanism analysis. Investment utilization coefficient will directly affect the pollutant handling capacity, here we take investment utilization coefficient of phosphogypsum as an example (Fig. S16a). Changes in investment utilization coefficient will inevitably lead to changes in the amount of phosphogypsum treatment, thereby affecting the corresponding changes in non-phosphorus product yield, resulting in non-phosphorus product output changes (Fig. S16b). While the cumulative storage of phosphogypsum depends on the difference between the amount of production and treatment, so the change in the amount of treatment does not directly affect the cumulative storage (Fig S16c). Changes in public satisfaction are subject to changes in all the cumulative discharge of pollutants in WPPAP and TPPAP, so a single change in phosphogypsum will just slightly affect public satisfaction (Fig S16d). Therefore, the sensitivity analysis shows that the model is sound and can be applied to additional strategy simulations.

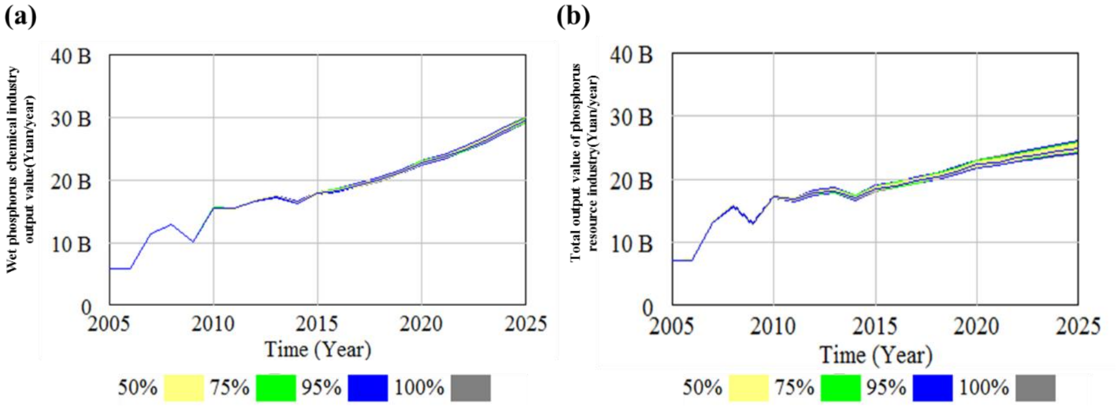

Fig. S15 Sensitivity analysis of environmental protection investment ratio of WPPAP. (a) Total output value of WPPAP industry; (b) Total output value of phosphorus resource industry (including non-phosphate products and economic losses). The environmental protection investment ratio of WPPAP was defined as a stochastic uniform distribution, ranging from 0.005 to 0.015, and the model was run 200 times to examine the distribution of the results. In the result, the yellow area represents 50% of the running results that are distributed in this range. The green area, blue area and gray area represent 75%, 90%, 100% of the running results those are distributed in this range, respectively.

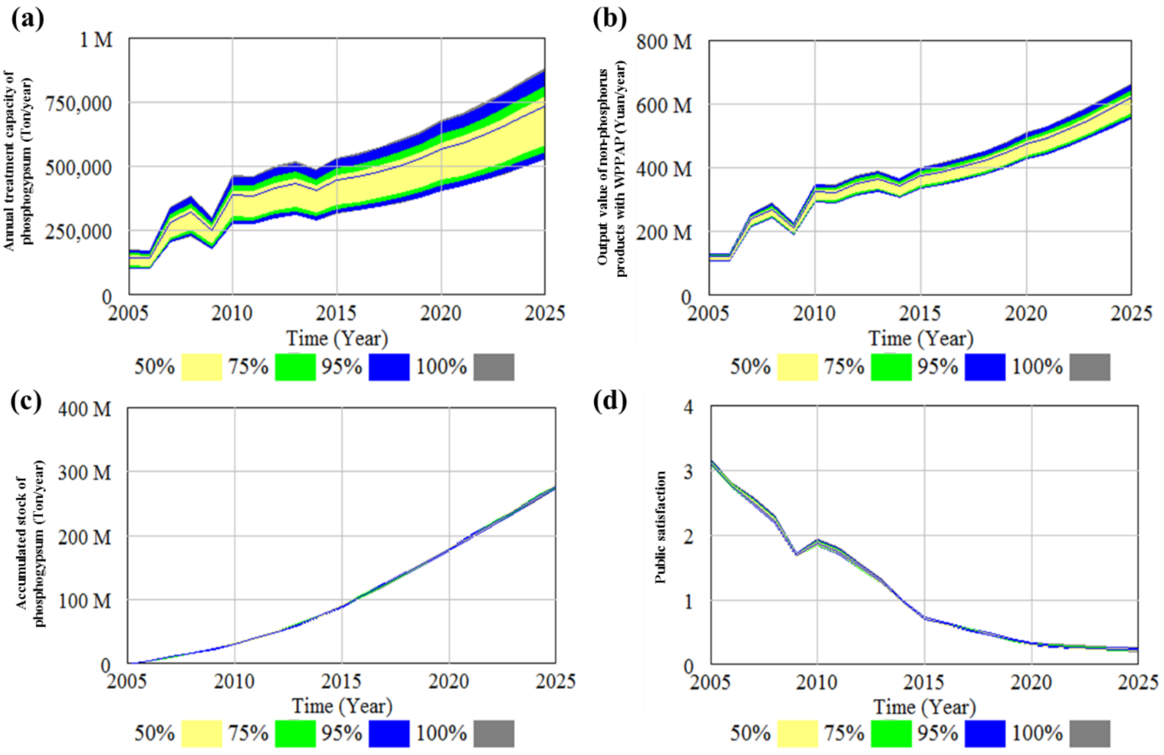

Fig. S16 Sensitivity analysis of investment utilization coefficient. (a) Annual treatment capacity of phosphogypsum; (b) Output value of non-phosphorus products with WPPAP; (c) Accumulated stock of

phosphogypsum; (d) Public satisfaction. The investment utilization coefficient was defined as a stochastic uniform distribution ranging from 150 to 200, and the model was run 200 times to examine the distribution of the results. In the run result, the yellow area represents 50% of the running results that are distributed in this range. The green area, blue area and gray area represent 75%, 90%, 100% of the running results those are distributed in this range, respectively.

4 Rural Internet penetration rate

The data were mainly collected from the source the "China Statistical Yearbook 2006-2014" and network public project data.

Using the trend extrapolation method and extrapolating the trend of 2014-2025 from the average growth rate of 2007-2020, the average growth rate of rural Internet users in 2007-2014 was 22.7% and that of rural users in 2014 was 28.8%. According to the relevant planning of the State Council, in 2020, the ratio of rural areas (administrative villages) open to Internet business will reach 98%; it is expected to reach 100% in 2021. The rural Internet penetration rate is shown in Figure S17.

The rural Internet penetration rate=WITH LOOKUP (Time, ([(2014,0)-(2025,1)], (2014,0.29), (2015,0.35), (2016,0.43), (2017,0.53), (2018,0.65), (2019,0.8), (2020,0.98), (2021,1), (2022,1), (2023,1), (2024,1), (2025,1))).

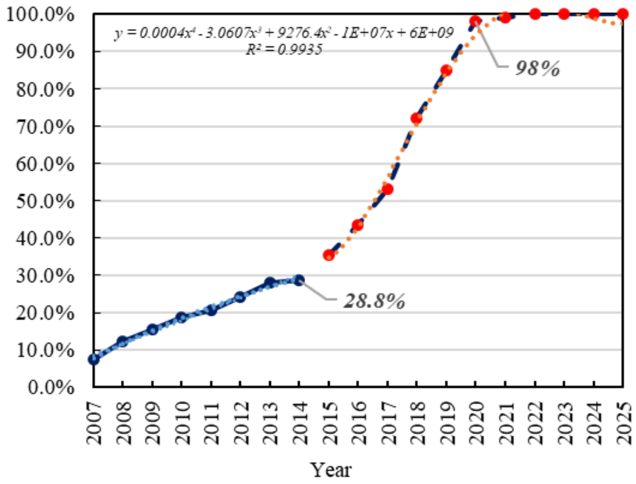

Fig.S17 Prediction curve of rural Internet penetration.

5 Agricultural big data support rate

According to the actual situation, the agricultural big data support rate is shown in Figure S18. The starting point of 2014 is 0.01%, and the end point of 2025 is 100%. The formula is as follows, where y is the agricultural big data support rate, x is the time, and $R^2 = 0.9981$:

$$y = 0.0012x^3 - 0.015x^2 + 0.093x - 0.0926 \text{ (Eq. S1)}$$

The agricultural big data support rate= WITH LOOKUP (Time-2014, ([(0,0)-(11,1)], (0,0), (2.14824,0.0747331), (4.89176,0.192171), (7.35059,0.355872), (7.55765,0.377224), (9.05882,0.55516), (9.96471,0.733096), (11,1)))

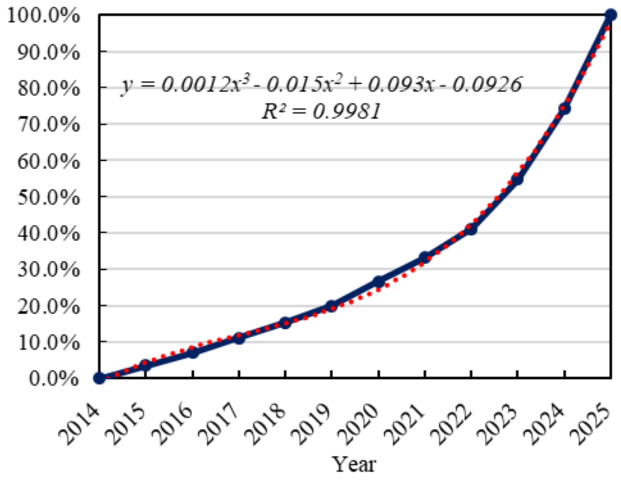

Fig.S18 Trend of the agricultural big data support rate over time.

Tables

The range of parameters in different scenarios.

Table S13 Range of the main variables and parameters in scenario design.

Variable and parameter	Ranges	Equation
Agricultural big data support rate	0-1	Agricultural big data support rate = WITH LOOKUP(Time-2014)
Rural Internet penetration rate	0-1	Rural Internet penetration rate = WITH LOOKUP(Time)
Agricultural Internet platform coverage	0-1	Agricultural Internet platform coverage = (ARCSIN(Regional technical progress index))*(1.185+ Regional business model change degree)* Internet control parameters
Relying on agricultural value-added service platform coverage	0-1	Relying on agricultural value-added services platform coverage = DELAY1(IF THEN ELSE(Agricultural Internet platform coverage <1, Agricultural Internet platform coverage * Online service improvement factor, 1), 0.5)
Control parameters of traditional fertilizers for soil testing and fertilization	0 or 1	0 means that the control does not work, and 1 means that the control is in effect.
Control Parameter of willingness to produce new fertilizer of enterprise	0 or 1	0 means that the control does not work, and 1 means that the control is in effect.
Demand for new fertilizer customization	0-1	Demand for new fertilizer customization =0.308* Relying on agricultural value-added services platform coverage ^2+0.01*EXP(Rural Internet penetration rate)+0.727* Agricultural big data support rate ^2

Demand for new fertilizer customization

Table S14 Variance analysis of demand for new fertilizer customization.

Source	Sum of square	Degree of freedom	Mean square
Regression analysis	1.141	3	0.380
Residual analysis	0.001	9	0.000
Total uncorrected	1.141	12	-
Total corrected	0.735	11	-

Figures

Case study: phosphorous fertilizer industry in Guizhou Province, China.

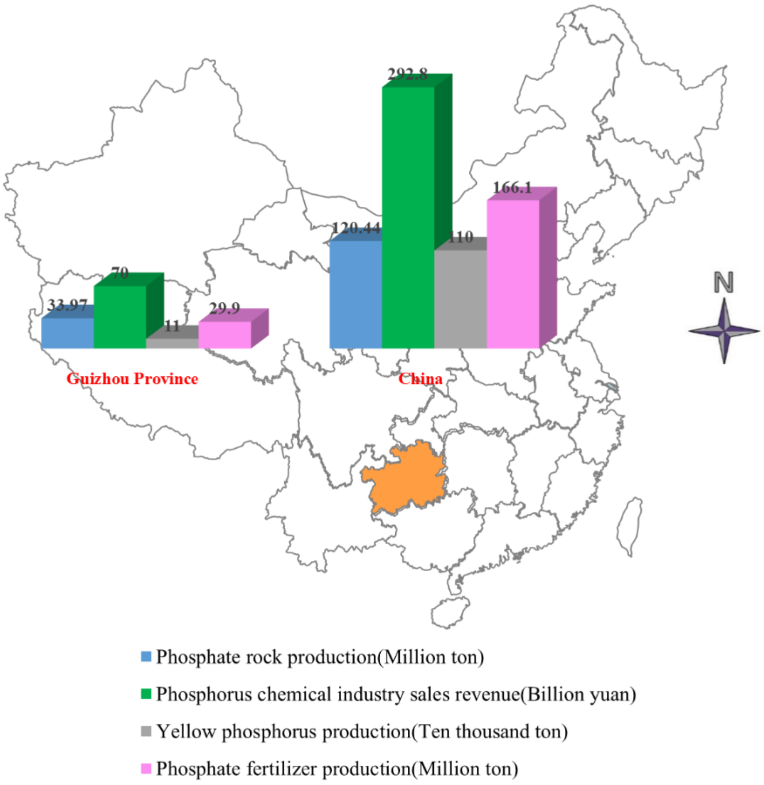

Fig. S19 Location of Guizhou Province in China and the development of the phosphorus chemicals industry.

Indirect benefit analysis at the government level

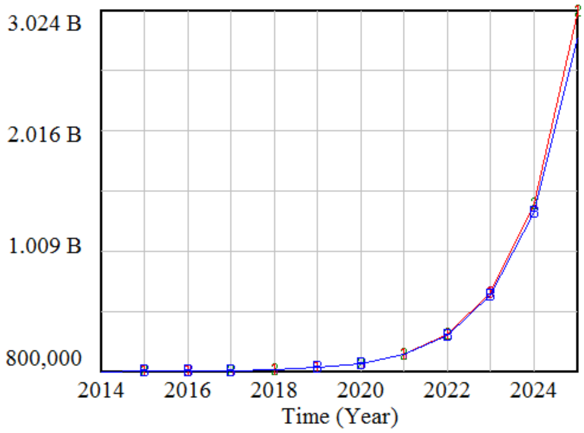

Fig.S20 Water conservation curve. Curves with B, 1, 2, and 3 represent the baseline scenario S0 and scenarios S1-3, respectively. The unit of the vertical axis is billion tons.

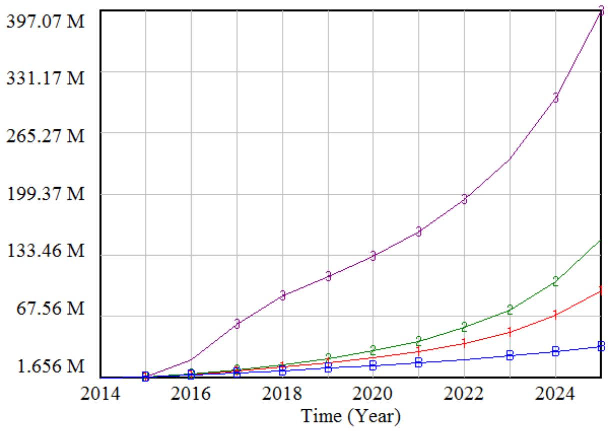

Fig.S21 Value-added of coal savings under different scenarios. Curves with B, 1, 2, and 3 represent the baseline scenario S0 and scenarios S1-3, respectively. The unit of the vertical axis is million yuan.

References

1 Luo, Z., Ma, S., Hu, S., Chen, D. 2017 Towards the sustainable development of the regional phosphorus resources industry in China: A system dynamics approach. *Resources, Conservation and Recycling*. 126, 186-197. (https://doi.org/10.1016/j.resconrec.2017.07.018)

2 Ma, S., Hu, S., Chen, D., Feng, Y. 2015 A Study of the Sustainable Development of China's Phosphorus Resources Industry Based on System Dynamics. *Computer Aided Chemical Engineering*. 37, 2051-2056.

3 Arrow, K., Bolin, B., Costanza, R., Dasgupta, P., Folke, C., Holling, C. S., Jansson, B. O., Levin, S., Mäler, K. G., Perrings, C.*, et al.* 1995 Economic growth, carrying capacity, and the environment. *Science*. 268, 520. (https://doi.org/10.1017/S1355770X00000413)

4 Wills, B. A., Napier-Munn, T. 2015 *Wills' mineral processing technology: an introduction to the practical aspects of ore treatment and mineral recovery*. Butterworth-Heinemann.

5 Liu, Y. 2005 Phosphorus resources at home & abroad, and the current s ituation of their exploitation & utilization (in Chinese). *Phosphate & Compound Fertilizer*. 5, 000.

6 Shi, Y., Wang, L., Liu, S., Nie, H. 2008 Development of chemical fertilizer industry and its effect on agriculture of China (in Chinese). *Acta Pedologica Sinica*. 5, 012.

7 Donatello, S., Tong, D., Cheeseman, C. R. 2010 Production of technical grade phosphoric acid from incinerator sewage sludge ash (ISSA). *Waste management*. 30, 1634-1642. (https://doi.org/10.1016/j.wasman.2010.04.009)

8 Hannachi, A., Habaili, D., Chtara, C., Ratel, A. 2007 Purification of wet process phosphoric acid by solvent extraction with TBP and MIBK mixtures. *Separation and purification technology*. 55, 212-216. (https://doi.org/10.1016/j.seppur.2006.12.014)

9 Ohzuku, T., Brodd, R. J. 2007 An overview of positive-electrode materials for advanced lithium-ion batteries. *Journal of Power Sources*. 174, 449-456. (https://doi.org/10.1016/j.jpowsour.2007.06.154)

10 Xu, K. 2004 Nonaqueous liquid electrolytes for lithium-based rechargeable batteries. *Chemical reviews*. 104, 4303-4418. (10.1021/cr030203g)

11 Joseph, P., Ebdon, J. R. 2009 Phosphorus-based flame retardants. *Fire Retardancy of Polymeric Materials*. 2,

12 Zhang, G. 2008 The development trend of feed-grade phosphate for MDCP (in Chinese). *Phosphate & Compound Fertilizer*. 1, 015.

13 Boudreau, J. W. 1983 Economic considerations in estimating the utility of human resource productivity improvement programs. *Personnel Psychology*. 36, 551-576. (https://doi.org/10.1111/j.1744-6570.1983.tb02235.x)

14 Tayibi, H., Choura, M., López, F. A., Alguacil, F. J., López-Delgado, A. 2009 Environmental impact and management of phosphogypsum. *Journal of Environmental Management*. 90, 2377-2386. (https://doi.org/10.1016/j.jenvman.2009.03.007)

15 Singh, M. 2002 Treating waste phosphogypsum for cement and plaster manufacture. *Cement and Concrete Research*. 32, 1033-1038. (https://doi.org/10.1016/S0008-8846(02)00723-8)

16 Reijnders, L. 2007 Cleaner phosphogypsum, coal combustion ashes and waste incineration ashes for application in building materials: A review. *Building and Environment*. 42, 1036-1042. (https://doi.org/10.1016/j.buildenv.2005.09.016)

17 Mori, Y., Kyotani, T., Shinohara, T. Method and apparatus for treating a waste gas containing fluorine-containing compounds. Google Patents 2005.

18 Barber, J. C. Remediation of soil polluted with phosphorus-containing wastes. Google Patents 2001.

19 Yan, M. 2009 Way of innovation and recycling economy for development of phosphorus chemical industry (I): reclaiming, purification, and utilization of tail gas of yellow phosphorus (in Chinese). *Inorganic Chemicals Industry*. 10, 000.

20 Lee, P. C., Su, H. N. 2010 Investigating the structure of regional innovation system research through keyword co-occurrence and social network analysis. *Innovation*. 12, 26-40. (https://doi.org/10.5172/impp.12.1.26)

21 Wong, P. K., Ho, Y. P., Singh, A. 2010 Industrial cluster development and innovation in Singapore. In *From agglomeration to innovation*. (ed.^eds. pp. 50-116: Springer.

22 Singh, J. 2008 Distributed R&D, cross-regional knowledge integration and quality of innovative output. *Research Policy*. 37, 77-96. (https://doi.org/10.1016/j.respol.2007.09.004)

23 Geyskens, I., Steenkamp, J.-B. E. 2000 Economic and social satisfaction: measurement and relevance to marketing channel relationships. *Journal of Retailing*. 76, 11-32. (https://doi.org/10.1016/S0022-4359(99)00021-4)

24 del Bosque Rodríguez, I. R., Agudo, J. C., Gutiérrez, H. S. M. 2006 Determinants of economic and social satisfaction in manufacturer–distributor relationships. *Industrial Marketing Management*. 35, 666-675. (https://doi.org/10.1016/j.indmarman.2005.05.006)

25 Jaumotte, F., Pain, N. 2005 An overview of public policies to support innovation. (https://doi.org/10.1787/707375561288)

26 Liu, H., Benoit, G., Liu, T., Liu, Y., Guo, H. 2015 An integrated system dynamics model developed for managing lake water quality at the watershed scale. *Journal of environmental management*. 155, 11-23. (https://doi.org/10.1016/j.jenvman.2015.02.046)
